# Changes in in-hospital mortality in the first wave of COVID-19: a multicentre prospective observational cohort study using the WHO Clinical Characterisation Protocol UK

**DOI:** 10.1016/S2213-2600(21)00175-2

**Published:** 2021-07

**Authors:** Annemarie B Docherty, Rachel H Mulholland, Nazir I Lone, Christopher P Cheyne, Daniela De Angelis, Karla Diaz-Ordaz, Cara Donegan, Thomas M Drake, Jake Dunning, Sebastian Funk, Marta García-Fiñana, Michelle Girvan, Hayley E Hardwick, Janet Harrison, Antonia Ho, David M Hughes, Ruth H Keogh, Peter D Kirwan, Gary Leeming, Jonathan S Nguyen Van-Tam, Riinu Pius, Clark D Russell, Rebecca G Spencer, Brian DM Tom, Lance Turtle, Peter JM Openshaw, J Kenneth Baillie, Ewen M Harrison, Malcolm G Semple, ISARIC4C Investigators, J Kenneth Baillie, Malcolm G Semple, Peter JM Openshaw, Gail Carson, Beatrice Alex, Benjamin Bach, Wendy S Barclay, Debby Bogaert, Meera Chand, Graham S Cooke, Annemarie B Docherty, Jake Dunning, Ana da Silva Filipe, Tom Fletcher, Christopher A Green, Ewen M Harrison, Julian A Hiscox, Antonia YW Ho, Peter W Horby, Samreen Ijaz, Say Khoo, Paul Klenerman, Andrew Law, Wei Shen Lim, Alexander J Mentzer, Laura Merson, Alison M Meynert, Mahdad Noursadeghi, Shona C Moore, Massimo Palmarini, William A Paxton, Georgios Pollakis, Nicholas Price, Andrew Rambaut, David L Robertson, Clark D Russell, Vanessa Sancho-Shimizu, Janet T Scott, Thushan de Silva, Louise Sigfrid, Tom Solomon, Shiranee Sriskandan, David Stuart, Charlotte Summers, Richard S Tedder, Emma C Thomson, AA Roger Thompson, Ryan S Thwaites, Lance CW Turtle, Rishi K Gupta, Carlo Palmieri, Maria Zambon, Hayley Hardwick, Chloe Donohue, Ruth Lyons, Fiona Griffiths, Wilna Oosthuyzen, Lisa Norman, Riinu Pius, Thomas M Drake, Cameron J Fairfield, Stephen R Knight, Kenneth A Mclean, Derek Murphy, Catherine A Shaw, Jo Dalton, Michelle Girvan, Egle Saviciute, Stephanie Roberts, Janet Harrison, Laura Marsh, Marie Connor, Sophie Halpin, Clare Jackson, Carrol Gamble, Gary Leeming, Andrew Law, Murray Wham, Sara Clohisey, Ross Hendry, James Scott-Brown, William Greenhalf, Victoria Shaw, Sarah E McDonald, Seán Keating, Katie A Ahmed, Jane A Armstrong, Milton Ashworth, Innocent G Asiimwe, Siddharth Bakshi, Samantha L Barlow, Laura Booth, Benjamin Brennan, Katie Bullock, Benjamin WA Catterall, Jordan J Clark, Emily A Clarke, Sarah Cole, Louise Cooper, Helen Cox, Christopher Davis, Oslem Dincarslan, Chris Dunn, Philip Dyer, Angela Elliott, Anthony Evans, Lorna Finch, Lewis WS Fisher, Terry Foster, Isabel Garcia-Dorival, William Greenhalf, Philip Gunning, Catherine Hartley, Rebecca L Jensen, Christopher B Jones, Trevor R Jones, Shadia Khandaker, Katharine King, Robyn T Kiy, Chrysa Koukorava, Annette Lake, Suzannah Lant, Diane Latawiec, Lara Lavelle-Langham, Daniella Lefteri, Lauren Lett, Lucia A Livoti, Maria Mancini, Sarah McDonald, Laurence McEvoy, John McLauchlan, Soeren Metelmann, Nahida S Miah, Joanna Middleton, Joyce Mitchell, Shona C Moore, Ellen G Murphy, Rebekah Penrice-Randal, Jack Pilgrim, Tessa Prince, Will Reynolds, P. Matthew Ridley, Debby Sales, Victoria E Shaw, Rebecca K Shears, Benjamin Small, Krishanthi S Subramaniam, Agnieska Szemiel, Aislynn Taggart, Jolanta Tanianis-Hughes, Jordan Thomas, Erwan Trochu, Libby van Tonder, Eve Wilcock, J. Eunice Zhang, Lisa Flaherty, Nicole Maziere, Emily Cass, Alejandra Doce Carracedo, Nicola Carlucci, Anthony Holmes, Hannah Massey, Lee Murphy, Nicola Wrobel, Sarah McCafferty, Kirstie Morrice, Alan MacLean, Kayode Adeniji, Daniel Agranoff, Ken Agwuh, Dhiraj Ail, Erin L Aldera, Ana Alegria, Brian Angus, Abdul Ashish, Dougal Atkinson, Shahedal Bari, Gavin Barlow, Stella Barnass, Nicholas Barrett, Christopher Bassford, Sneha Basude, David Baxter, Michael Beadsworth, Jolanta Bernatoniene, John Berridge, Nicola Best, Pieter Bothma, David Chadwick, Robin Brittain-Long, Naomi Bulteel, Tom Burden, Andrew Burtenshaw, Vikki Caruth, David Chadwick, Duncan Chambler, Nigel Chee, Jenny Child, Srikanth Chukkambotla, Tom Clark, Paul Collini, Catherine Cosgrove, Jason Cupitt, Maria-Teresa Cutino-Moguel, Paul Dark, Chris Dawson, Samir Dervisevic, Phil Donnison, Sam Douthwaite, Ingrid DuRand, Ahilanadan Dushianthan, Tristan Dyer, Cariad Evans, Chi Eziefula, Chrisopher Fegan, Adam Finn, Duncan Fullerton, Sanjeev Garg, Sanjeev Garg, Atul Garg, Effrossyni Gkrania-Klotsas, Jo Godden, Arthur Goldsmith, Clive Graham, Elaine Hardy, Stuart Hartshorn, Daniel Harvey, Peter Havalda, Daniel B Hawcutt, Maria Hobrok, Luke Hodgson, Anil Hormis, Michael Jacobs, Susan Jain, Paul Jennings, Agilan Kaliappan, Vidya Kasipandian, Stephen Kegg, Michael Kelsey, Jason Kendall, Caroline Kerrison, Ian Kerslake, Oliver Koch, Gouri Koduri, George Koshy, Shondipon Laha, Steven Laird, Susan Larkin, Tamas Leiner, Patrick Lillie, James Limb, Vanessa Linnett, Jeff Little, Mark Lyttle, Michael MacMahon, Emily MacNaughton, Ravish Mankregod, Huw Masson, Elijah Matovu, Katherine McCullough, Ruth McEwen, Manjula Meda, Gary Mills, Jane Minton, Mariyam Mirfenderesky, Kavya Mohandas, Quen Mok, James Moon, Elinoor Moore, Patrick Morgan, Craig Morris, Katherine Mortimore, Samuel Moses, Mbiye Mpenge, Rohinton Mulla, Michael Murphy, Megan Nagel, Thapas Nagarajan, Mark Nelson, Matthew K O'Shea, Igor Otahal, Marlies Ostermann, Mark Pais, Selva Panchatsharam, Danai Papakonstantinou, Hassan Paraiso, Brij Patel, Natalie Pattison, Justin Pepperell, Mark Peters, Mandeep Phull, Stefania Pintus, Jagtur Singh Pooni, Frank Post, David Price, Rachel Prout, Nikolas Rae, Henrik Reschreiter, Tim Reynolds, Neil Richardson, Mark Roberts, Devender Roberts, Alistair Rose, Guy Rousseau, Brendan Ryan, Taranprit Saluja, Aarti Shah, Prad Shanmuga, Anil Sharma, Anna Shawcross, Jeremy Sizer, Manu Shankar-Hari, Richard Smith, Catherine Snelson, Nick Spittle, Nikki Staines, Tom Stambach, Richard Stewart, Pradeep Subudhi, Tamas Szakmany, Kate Tatham, Jo Thomas, Chris Thompson, Robert Thompson, Ascanio Tridente, Darell Tupper-Carey, Mary Twagira, Andrew Ustianowski, Nick Vallotton, Lisa Vincent-Smith, Shico Visuvanathan, Alan Vuylsteke, Sam Waddy, Rachel Wake, Andrew Walden, Ingeborg Welters, Tony Whitehouse, Paul Whittaker, Ashley Whittington, Padmasayee Papineni, Meme Wijesinghe, Martin Williams, Lawrence Wilson, Sarah Sarah, Stephen Winchester, Martin Wiselka, Adam Wolverson, Daniel G Wooton, Andrew Workman, Bryan Yates, Peter Young

**Affiliations:** a Centre for Medical Informatics, University of Edinburgh, Edinburgh, UK; b The Breathe Hub, University of Edinburgh, Edinburgh, UK; c Centre for Population Health Sciences, University of Edinburgh, Edinburgh, UK; d The Usher Institute, Queen's Medical Research Institute, University of Edinburgh, Edinburgh, UK; e Roslin Institute, University of Edinburgh, Edinburgh, UK; f Department of Health Data Science, Institute of Population Health, University of Liverpool, Liverpool, UK; g Health Protection Research Unit in Emerging and Zoonotic Infections, Institute of Infection, Veterinary and Ecological Sciences, Faculty of Health and Life Sciences, University of Liverpool, Liverpool, UK; h Liverpool Clinical Trials Centre, University of Liverpool, Liverpool, UK; i MRC Biostatistics Unit, University of Cambridge, Cambridge, UK; j London School of Hygiene & Tropical Medicine, London, UK; k National Heart and Lung Institute, Faculty of Medicine, Imperial College London, London, UK; l MRC University of Glasgow Centre for Virus Research, Glasgow, UK; m Division of Epidemiology and Public Health, School of Medicine, University of Nottingham, Nottingham, UK; n Department of Respiratory Medicine, Alder Hey Children's Hospital, Liverpool, UK

## Abstract

**Background:**

Mortality rates in hospitalised patients with COVID-19 in the UK appeared to decline during the first wave of the pandemic. We aimed to quantify potential drivers of this change and identify groups of patients who remain at high risk of dying in hospital.

**Methods:**

In this multicentre prospective observational cohort study, the International Severe Acute Respiratory and Emerging Infections Consortium WHO Clinical Characterisation Protocol UK recruited a prospective cohort of patients with COVID-19 admitted to 247 acute hospitals in England, Scotland, and Wales during the first wave of the pandemic (between March 9 and Aug 2, 2020). We included all patients aged 18 years and older with clinical signs and symptoms of COVID-19 or confirmed COVID-19 (by RT-PCR test) from assumed community-acquired infection. We did a three-way decomposition mediation analysis using natural effects models to explore associations between week of admission and in-hospital mortality, adjusting for confounders (demographics, comorbidities, and severity of illness) and quantifying potential mediators (level of respiratory support and steroid treatment). The primary outcome was weekly in-hospital mortality at 28 days, defined as the proportion of patients who had died within 28 days of admission of all patients admitted in the observed week, and it was assessed in all patients with an outcome. This study is registered with the ISRCTN Registry, ISRCTN66726260.

**Findings:**

Between March 9, and Aug 2, 2020, we recruited 80 713 patients, of whom 63 972 were eligible and included in the study. Unadjusted weekly in-hospital mortality declined from 32·3% (95% CI 31·8–32·7) in March 9 to April 26, 2020, to 16·4% (15·0–17·8) in June 15 to Aug 2, 2020. Reductions in mortality were observed in all age groups, in all ethnic groups, for both sexes, and in patients with and without comorbidities. After adjustment, there was a 32% reduction in the risk of mortality per 7-week period (odds ratio [OR] 0·68 [95% CI 0·65–0·71]). The higher proportions of patients with severe disease and comorbidities earlier in the first wave (March and April) than in June and July accounted for 10·2% of this reduction. The use of respiratory support changed during the first wave, with gradually increased use of non-invasive ventilation over the first wave. Changes in respiratory support and use of steroids accounted for 22·2%, OR 0·95 (0·94–0·95) of the reduction in in-hospital mortality.

**Interpretation:**

The reduction in in-hospital mortality in patients with COVID-19 during the first wave in the UK was partly accounted for by changes in the case-mix and illness severity. A significant reduction in in-hospital mortality was associated with differences in respiratory support and critical care use, which could partly reflect accrual of clinical knowledge. The remaining improvement in in-hospital mortality is not explained by these factors, and could be associated with changes in community behaviour, inoculum dose, and hospital capacity strain.

**Funding:**

National Institute for Health Research and the Medical Research Council.

## Introduction

There is growing evidence that mortality from COVID-19 declined during the first wave of the pandemic in the UK, both in hospital and in the community.^1^, ^2^, ^3^, ^4^, ^5^ One explanation for this decline could be that the case-mix of patients presenting to hospital changed across time towards a younger and less comorbid demographic, who were at lower risk of dying than were patients at the start of the first wave. The national UK lockdown and effective shielding measures in susceptible at-risk populations could have reduced transmission of the virus during the course of the first wave.^6^ Additionally, easier access to testing and advice about seeking medical help might have resulted in earlier presentation to hospital. Familiarity with the virus and clinical course could have also led to better management of patients through improved ward and intensive care unit (ICU) care.^7^, ^8^ Corticosteroids have been shown in trials to reduce mortality in patients with severe COVID-19, and there was an increase in the use of corticosteroids during the course of the first wave.^9^, ^10^

**Research in context**

**Evidence before this study**

We searched PubMed on March 19, 2021, using the search terms “SARS-CoV-2” OR “COVID-19” AND “mortality” AND “hospital”. We searched for primary research articles documenting changes in COVID-19-related mortality in hospitals over time published between March 1, 2020, and March 19, 2021, with no language restrictions. Of the 202 articles identified, most focused on risk factors for mortality, and we found only four studies that documented changes in mortality over time. None of these four studies explored the potential reasons for why COVID-19-related mortality rates in hospitals are declining beyond patient demographics. Understanding changes in mortality rates over time will help policy makers identify evolving risk and strategies to manage this evolving risk, and make broader decisions about public health interventions.

**Added value of this study**

To our knowledge, this is the first study to explore changes in COVID-19-related mortality in the context of patient demographics, severity of illness at presentation to hospital, and treatments received. Mortality in hospitalised patients at the beginning of the first wave (ie, between March and April, 2020) of the pandemic was extremely high. Patients who were admitted to hospital in March and early April, 2020, were substantially more unwell at presentation than patients who were admitted in later months (June and July 2020). Mortality declined in all age groups, in all ethnic groups, in men and women, and in patients with and without comorbidities, over and above contributions from declining illness severity. After adjustment for these variables, a fifth of the reduction in mortality was explained by changes in the use of respiratory support and steroid treatment. However, mortality was persistently high in patients who required invasive mechanical ventilation, and in those who received non-invasive ventilation outside of critical care.

**Implications of all the available evidence**

The observed reduction in COVID-19-related hospital mortality was greater than would have been expected from changes observed in both case-mix and illness severity. This reduction in mortality was partly attributable to differences in respiratory support and critical care use, which could reflect, in part, accrual of clinical knowledge. In addition, the introduction of community policies, such as the wearing of masks, physical distancing, the shielding of susceptible patients, and the UK lockdown, potentially resulted in people being exposed to less virus. We found that the decrease in mortality varied depending on the level of respiratory support received. Patients receiving invasive mechanical ventilation have persistently high mortality rates, albeit with a changing case-mix, and further research should target this group of patients. Severe COVID-19 has primarily affected older (ie, those aged >70 years) people in the UK. It is essential to ensure that patients and their families remain at the centre of decision making, and that we continue to use an individualised approach to their treatment and care.

The International Severe Acute Respiratory and Emerging Infections Consortium (ISARIC) WHO Clinical Characterisation Protocol UK (CCP-UK)^11^ was activated on Jan 17, 2020, to recruit patients with COVID-19 admitted to a network of hospitals in England, Scotland, and Wales.^12^ ISARIC has been prepared for outbreaks such as COVID-19 for the past 8 years, with the aim of providing data and samples for near real-time analysis.^11^ During the COVID-19 outbreak, analysis of the CCP-UK cohort in the first wave allowed development of the pragmatic International Severe Acute Respiratory and Emerging Infections Consortium Coronavirus Clinical Characterisation Consortium (ISARIC4C) Mortality Score for hospitalised patients with COVID-19 in readiness to aid clinical management decisions in the second wave.^13^

We aimed to use the ISARIC CCP-UK cohort to describe how in-hospital mortality changed over time in patients admitted to hospital with COVID-19. We aimed to explore potential drivers for these changes by assessing patient characteristics, illness severity, and treatment received during the hospital stay.

## Methods

### Study design and patients

We did a multicentre prospective observational cohort study at 247 acute general hospitals in England, Scotland, and Wales. We included adults (aged ≥18 years) from the ISARIC WHO CCP-UK cohort admitted to hospital during the first wave (between March 9 and Aug 2, 2020) with clinical signs and symptoms of COVID-19 or confirmed COVID-19 (by RT-PCR test) from assumed community acquired infection (appendix pp 2–3). The national strategy changed from containment to admission based on clinical need on March 12, 2020.^14^ Further information about the COVID-19 care model in the first wave in UK can be found in the appendix (pp 1–2). Community hospitals providing long-term treatment and residential mental health hospitals were excluded since patient populations were very different and were therefore not comparable. We excluded patients with nosocomial COVID-19 infection, who were defined as patients with onset of COVID-19 symptoms more than 5 days after they were admitted to hospital for a separate condition.

Ethical approval for data collection and analysis by the ISARIC4C was given by the South Central-Oxford C Research Ethics Committee in England (reference 13/SC/0149) and by the Scotland A Research Ethics Committee (reference 20/SS/0028). Under the Control of Patient Information notice 2020 for urgent public health research, processing of demographic and routine clinical data from medical records for research does not require patient consent in England and Wales.^15^ In Scotland, a waiver for consent was obtained from the Public Benefit and Privacy Panel for Health and Social Care. The ISARIC WHO CCP-UK study is registered with the ISRCTN Registry, ISRCTN66726260, and has been designated as an Urgent Public Health Research Study by the National Institute for Health Research (NIHR).

### Procedures

Data were extracted (cutoff date Sept 17, 2020) from routine health-care records and recorded in case report forms on the REDCap database. The ISARIC 4C Investigators collected information on key variables including patient characteristics, illness severity, level of respiratory support, COVID-19-specific treatments, and in-hospital mortality. Patient characteristics included age group (<50 years, 50–69 years, 70–79 years, and ≥80 years, consistent with previous ISARIC4C studies); sex (male or female); self-reported ethnicity (south Asian, east Asian, Black, other minority ethnic group, and White);^16^ index of multiple deprivation (derived from individual patient postal codes); health worker status (yes or no); and number of comorbidities (none, one, or two or more), measured by use of a modified Charlson Comorbidity Index (see appendix [p 3] for further details).

Severity of illness at hospital admission (or within 24 h of admission) was assessed by use of the following physiological components of the ISARIC4C Mortality Score:^13^ respiratory rate (breaths per min); peripheral oxygen saturation on room air (SpO_2_); Glasgow coma scale score; serum urea concentration (mmol/L); and C-reactive protein concentration (mg/L). To capture patterns in access to hospital treatment, we calculated the number of days from symptom onset to hospital admission.

Patients were categorised as managed on the ward only or in critical care (ie, in the ICU or high dependency unit [HDU]) at any time (see appendix [pp 1–4] for more information on level of respiratory support). Maximum level of respiratory support received was classified as no respiratory support, oxygen (face mask, nasal cannulae, or high-flow nasal oxygen), non-invasive ventilation, and invasive mechanical ventilation.

For COVID-19-specific treatments, we only recorded whether patients had received corticosteroids (dexamethasone, hydrocortisone, methylprednisolone, or prednisolone), as these were the only treatments with recognised mortality benefit for COVID-19 in randomised controlled trials.^9^, ^17^

The main exposure of interest was the week of admission to hospital, defined as the International Organization for Standardization date week (ie, the ordinal week of the year). To facilitate comparison across time periods, week of admission to hospital was also categorised into three equal time periods (time period 1 included weeks 11–17 [from March 9 to April 26, 2020]; time period 2 included weeks 18–24 [from April 27 to June 14, 2020]; and time period 3 included weeks 25–31 [from June 15 to Aug 2, 2020]).

### Outcomes

The primary outcome was the weekly in-hospital mortality at 28 days, defined as the proportion of patients who had died within 28 days of admission of all patients admitted in the observed week. Mortality was defined as an outcome of death or discharge to palliative care. The 28-day threshold aligns with the Public Health England definition of death due to COVID-19.^18^ We included all patients who were admitted to hospital at least 6 weeks before data analysis to allow for 28 days follow-up.

Secondary outcomes were changes in patient demographics and illness severity in patients managed on the ward and in critical care units. Within critical care, we looked separately at changes in the proportion of patients receiving oxygen only, non-invasive ventilation, and invasive mechanical ventilation. Within ward care, we looked at changes in the proportion of patients receiving no respiratory support, oxygen only, and non-invasive ventilation. We performed two sensitivity analyses: a complete case analysis in which only patients with outcomes (survived, died, or ongoing care) were included and an analysis in which patients with missing outcomes were assumed to be survivors.

### Statistical analysis

Continuous data are presented as means (SDs) or medians (IQRs) depending on the distribution. Categorical data are presented as percentage frequencies. For univariable comparisons, we used Welch's *t* test, ANOVA, Mann-Whitney *U* test, or Kruskal-Wallis tests, according to data distribution. Categorical data were compared by use of χ^2^ tests. Counts and proportions for each of the exposure variables were calculated across the three time periods. The proportion of patients admitted each week and weekly in-hospital mortality at 28 days were stratified by each explanatory variable of interest. 95% CIs for these proportions were calculated by use of the exact method.

There were missing data because of the challenges of real-time data collection during a pandemic. Missing data are reported in the results section and in appendix (pp 37–39), and patterns of missing data were explored. Missing data for number of comorbidities were classified as none, for health-care worker status were classified as no, and for respiratory support received (ie, oxygen, invasive mechanical ventilation, and non-invasive ventilation) were classified as none. For the primary analysis, multiple imputation with chained equations was done for missing markers of illness severity. Ten sets, each with ten iterations, were imputed by use of 35 explanatory variables including outcomes. We did graphical checks of convergence. All analyses were done by use of imputed datasets (see appendix [p 3] for further details).

For the primary outcome analysis, we excluded patients without an outcome date (classified as “survivors” in the sensitivity analysis results in the appendix [p 31). For the primary outcome, our modelling strategy was informed by a putative causal model (see the proposed directed acyclic graph in the appendix [p 26]). Using logistic regression, we specified three models exploring the association between admission week (as a continuous variable) and in-hospital mortality. A baseline model included adjustment for age, sex, and admitting hospital as a random effect. A second model accounted for known baseline confounders, including variables previously shown to be associated with in-hospital mortality (age, sex, number of comorbidities, index of multiple deprivation score, and severity of illness [respiratory rate, SpO_2_, Glasgow coma scale score, serum urea concentration, and C-reactive protein concentration]). In a third model, potential mediators were added to explore the effect of treatment (steroids and respiratory support, thus considering accrued clinical knowledge of respiratory support) on the association between week of admission and mortality (controlled direct-effect models). We extended this same model in a potential outcomes framework to perform a three-way decomposition mediation analysis using natural effects models.^19^ We sought to control confounding between exposure and outcome, exposure and mediator, and mediator and outcome, and we carefully considered potential mediator-outcome confounders influenced by the exposure. Using standard frequentist approaches, we imputed unobserved nested counterfactuals with an outcome model to accommodate our nominal mediator (respiratory support). Exposure-mediator interactions were explored and a joint model was used to incorporate steroid use. Robust SEs (based on a Sandwich estimator) were generated, and the results were presented as a proportion mediated on the risk difference scale.

For the secondary outcomes, to better understand patterns of mortality for different levels of respiratory support, time-series data were modelled with Bayesian generalised additive models to allow for easy incorporation of multiply imputed datasets (see appendix [p 3] for more details).

All statistical analyses were done in R (version 3.6.3) and Stan (rstan 2.21.2 and brms 2.14.4) with the tidyverse, finalfit, brms, mgcv, mice, medflex, gridExtra and cowplot packages. This study is registered with the ISRCTN Registry, ISRCTN66726260.

### Role of the funding source

The funders of the study had no role in study design, data collection, data analysis, data interpretation, or writing of the report.

## Results

**Figure 1:**
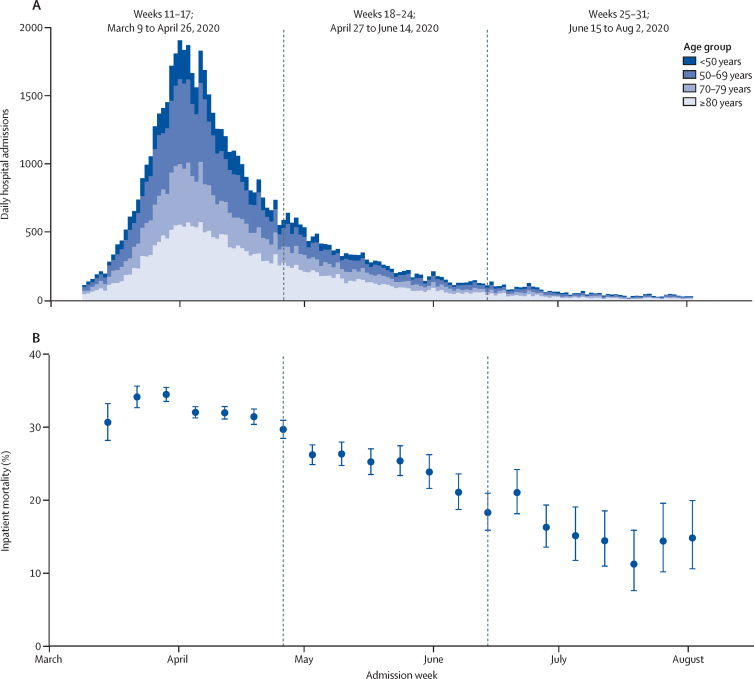
Hospital admissions and in-hospital mortality between March 9 and Aug 2, 2020 (A) Daily adult COVID-19 admissions by age. Cases are stacked by age group. (B) Weekly unadjusted mortality in adult inpatients admitted to hospital with COVID-19. Error bars represent 95% CIs, calculated by use of an exact method. Dashed lines indicate three equal time periods (weeks 11–17, 18–24, and 25–31).

Between March 9, and Aug 2, 2020, we recruited 80 713 patients from 247 acute hospitals in England, Scotland, and Wales, of whom 63 972 were eligible and included in the final cohort (table; appendix pp 5–7), representing approximately 48% of all hospital admissions in the UK during this time period.^1^ Admissions peaked in late March and in early April for all age groups, and steadily decreased until the end of the study period (figure 1A). A total of 15 864 (29·0%) of 54 632 patients managed on wards died within 28 days of hospital admission compared with 3317 (35·5%) of 9340 patients in critical care (appendix p 24). Of all 63 972 patients, 40 449 (63·2%) had a documented positive COVID-19 RT-PCR test result (appendix pp 6–7).

**Table:** Baseline characteristics of adult patients admitted to hospital with COVID-19, stratified by time (N=63 972)

		**Time period 1; weeks 11–17 (n=47 453)**	**Time period 2; weeks 18–24 (n=13 744)**	**Time period 3; weeks 25–31 (n=2775)**
**Patient characteristics**				
Country				
	England	43 326 (91·3%)	12 579 (91·5%)	2589 (93·3%)
	Scotland	2140 (4·5%)	534 (3·9%)	33 (1·2%)
	Wales	1987 (4·2%)	631 (4·6%)	153 (5·5%)
Age, years				
	Median	73·2 (58·6–83·4)	76·9 (62·2–85·8)	73·5 (57·5–83·4)
	Mean	70·1 (16·7)	72·6 (17·3)	69 (18·6)
Age group, years				
	<50	6261 (13·2%)	1663 (12·1%)	486 (17·5%)
	50–69	14 284 (30·1%)	3261 (23·7%)	734 (26·5%)
	70–79	10 675 (22·5%)	3015 (21·9%)	590 (21·3%)
	≥80	16 233 (34·2%)	5805 (42·2%)	965 (34·8%)
Sex				
	Female	19 837 (41·8%)	6659 (48·5%)	1353 (48·8%)
	Male	27 616 (58·2%)	7085 (51·5%)	1422 (51·2%)
Ethnicity				
	White	33 993 (71·6%)	10 832 (78·8%)	1922 (69·3%)
	South Asian	2279 (4·8%)	530 (3·9%)	296 (10·7%)
	East Asian	398 (0·8%)	41 (0·3%)	12 (0·4%)
	Black	2015 (4·2%)	236 (1·7%)	41 (1·5%)
	Other minority ethnic group	3340 (7%)	681 (5%)	229 (8·3%)
	Missing data	5428 (11·4%)	1424 (10·4%)	275 (9·9%)
Number of comorbidities				
	0	10 789 (22·7%)	2014 (14·7%)	507 (18·3%)
	1	11 389 (24·0%)	2463 (17·9%)	546 (19·7%)
	≥2	25 275 (53·3%)	9267 (67·4%)	1722 (62·1%)
Health worker		2419 (5·1%)	738 (5·4%)	77 (2·8%)
**Severity of illness**				
Asymptomatic		2446 (5·2%)	2117 (15·4%)	785 (28·3%)
Symptom onset, days*				
	Median	4·0 (0·0–7·0)	2·0 (0·0–7·0)	3·0 (0·0–7·0)
	Mean	5·0 (5·2)	4·1 (5·2)	4·1 (4·9)
Length of hospital stay, days				
	Median	8·0 (4·0–15·0)	9 (4·0–17·0)	8 (3·0–15·0)
	Mean	11·0 (12·6)	12·2 (11·9)	10·6 (9·7)
ISARIC4C mortality score				
	Low (0–3)	2056 (4·3%)	726 (5·3%)	196 (7·1%)
	Intermediate (4–8)	7469 (15·7%)	2020 (14·7%)	506 (18·2%)
	High (9–14)	16 509 (34·8%)	5416 (39·4%)	1042 (37·5%)
	Very high (≥15)	5055 (10·7%)	1321 (9·6%)	156 (5·6%)
	Missing data	16 364 (34·5%)	4261 (31%)	875 (31·5%)
Respiratory rate, breaths per min				
	<20	13 066 (27·5%)	5303 (38·6%)	1131 (40·8%)
	20–30	23 791 (50·1%)	6232 (45·3%)	1197 (43·1%)
	≥30	8433 (17·8%)	1670 (12·2%)	305 (11%)
	Missing data	2163 (4·6%)	539 (3·9%)	142 (5·1%)
Peripheral oxygen saturation on room air				
	≥92%	34 345 (72·4%)	10 970 (79·8%)	2259 (81·4%)
	<92%	10 667 (22·5%)	2224 (16·2%)	378 (13·6%)
	Missing data	2441 (5·1%)	550 (4%)	138 (5%)
Glasgow coma scale score				
	15	35 403 (74·6%)	10 857 (79%)	2323 (83·7%)
	<15	6645 (14%)	1911 (13·9%)	250 (9%)
	Missing data	5405 (11·4%)	976 (7·1%)	202 (7·3%)
Urea, mmol/L				
	<7	17 581 (37%)	5250 (38·2%)	1195 (43·1%)
	7–14	12 785 (26·9%)	3858 (28·1%)	757 (27·3%)
	>14	6572 (13·8%)	1869 (13·6%)	286 (10·3%)
	Missing data	10 515 (22·2%)	2767 (20·1%)	537 (19·4%)
C-reactive protein, mg/dL				
	<50	11 714 (24·7%)	4775 (34·7%)	1037 (37·4%)
	50–99	9400 (19·8%)	2397 (17·4%)	442 (15·9%)
	≥100	17 948 (37·8%)	3736 (27·2%)	684 (24·6%)
	Missing data	8391 (17·7%)	2836 (20·6%)	612 (22·1%)
**Respiratory support and treatments**				
Threshold of care				
	Critical care unit	7732 (16·3%)	1275 (9·3%)	333 (12%)
	Ward	39 721 (83·7%)	12 469 (90·7%)	2442 (88%)
Respiratory support				
	None	9314 (19·6%)	4780 (34·8%)	1184 (42·7%)
	Oxygen only	28 023 (59·1%)	7170 (52·2%)	1221 (44%)
	Non-invasive	5158 (10·9%)	1240 (9%)	272 (9·8%)
	Invasive	4958 (10·4%)	554 (4%)	98 (3·5%)
Steroids				
	Yes	7354 (15·5%)	2267 (16·5%)	910 (32·8%)
	No	37 162 (78·3%)	10 875 (79·1%)	1731 (62·4%)
	Missing data	2937 (6·2%)	602 (4·4%)	134 (4·8%)

Data are n (%), median (IQR), or mean (SD). Time period 1 was from March 9 to April 26, 2020; time period 2 was from April 27 to June 14, 2020; and time period 3 was from June 15 to Aug 2, 2020.

* Symptom onset summary statistics based on patients with symptoms up to 3 weeks before admission only. ISARIC4C=International Severe Acute Respiratory and Emerging Infections Consortium Coronavirus Clinical Characterisation Consortium.

**Figure 2:**
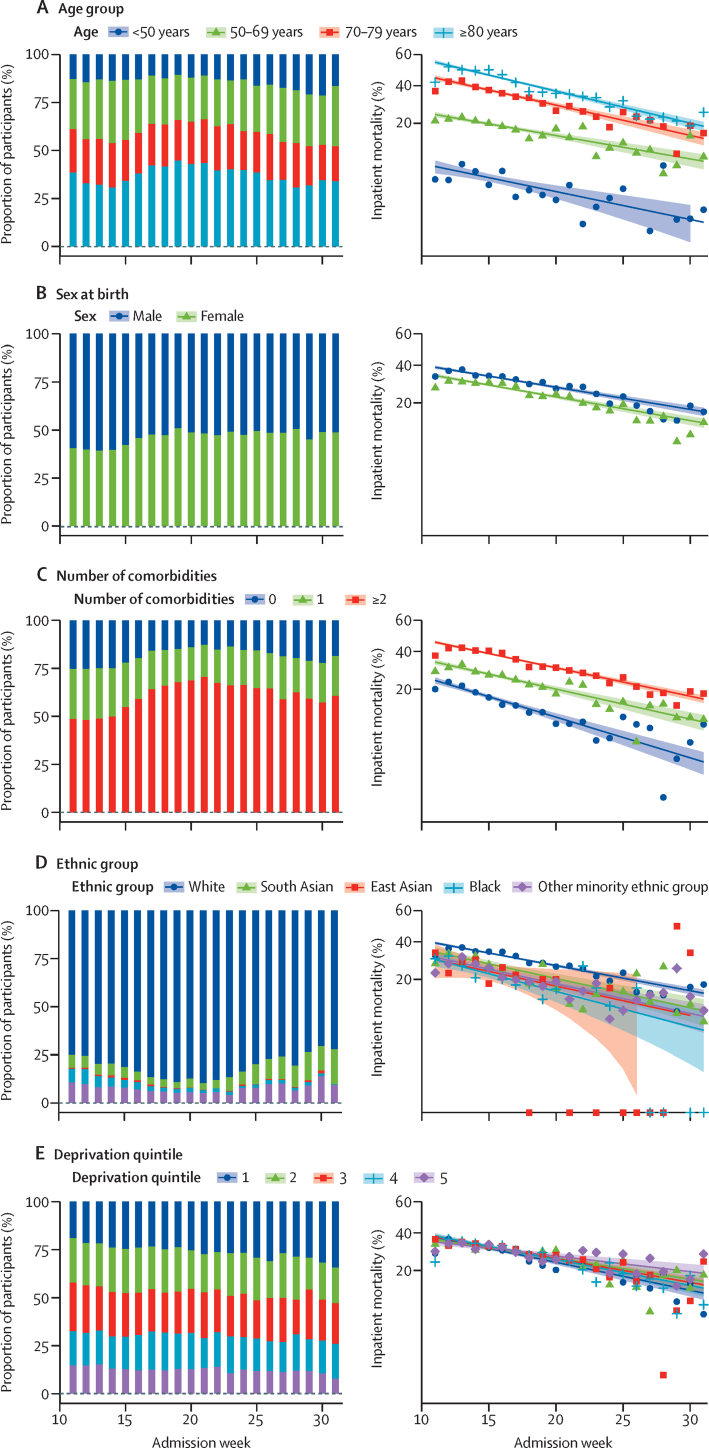
Proportion of adults admitted to hospital with COVID-19 and inpatient mortality between March 9 and Aug 2, 2020, stratified by age (A), sex at birth (B), number of comorbidities (C), ethnic group (D), and deprivation quintile (E) In A–E, all plots on the left show the proportion of adults admitted to hospital with COVID-19 by patient characteristics, and all plots on the right show unadjusted in-hospital mortality rates during the time period. In all plots on the left, the proportions of participants are stacked by characteristic. In E, the deprivation quintile ranges from 1 (most deprived) to 5 (least deprived). Missing data are excluded from the figure. Shaded areas represent 95% CIs.

Most patients (55 562 [86·9%] of 63 972) admitted during the first wave were aged 50 years or older. There was an increase in the proportion of younger people (ie, those aged <50 years) admitted over time (6261 [13·2%] of 47 453 patients in time period 1 *vs* 486 [17·5%] of 2775 patients in time period 3; table, figure 2A). There were initially more men admitted than women (27 616 [58·2%] of 47 453 patients in time period 1 were men), but the proportions of men and women were similar from mid-April to Aug 2, 2020 (figure 2B). The cohort was multimorbid, with 25 275 (53·3%) of 47 453 patients in time period 1 having two or more comorbidities. The proportion of patients with two or more comorbidities increased over time (table, figure 2C; appendix p 7). Most patients were White, with an increasing proportion of south Asian patients and a decreasing proportion of patients from Black ethnic groups over time (figure 2D). In all time periods, the highest proportion of patients were in the most deprived quintile, and the proportion of patients in this deprivation quintile increased over time (figure 2E).

**Figure 3:**
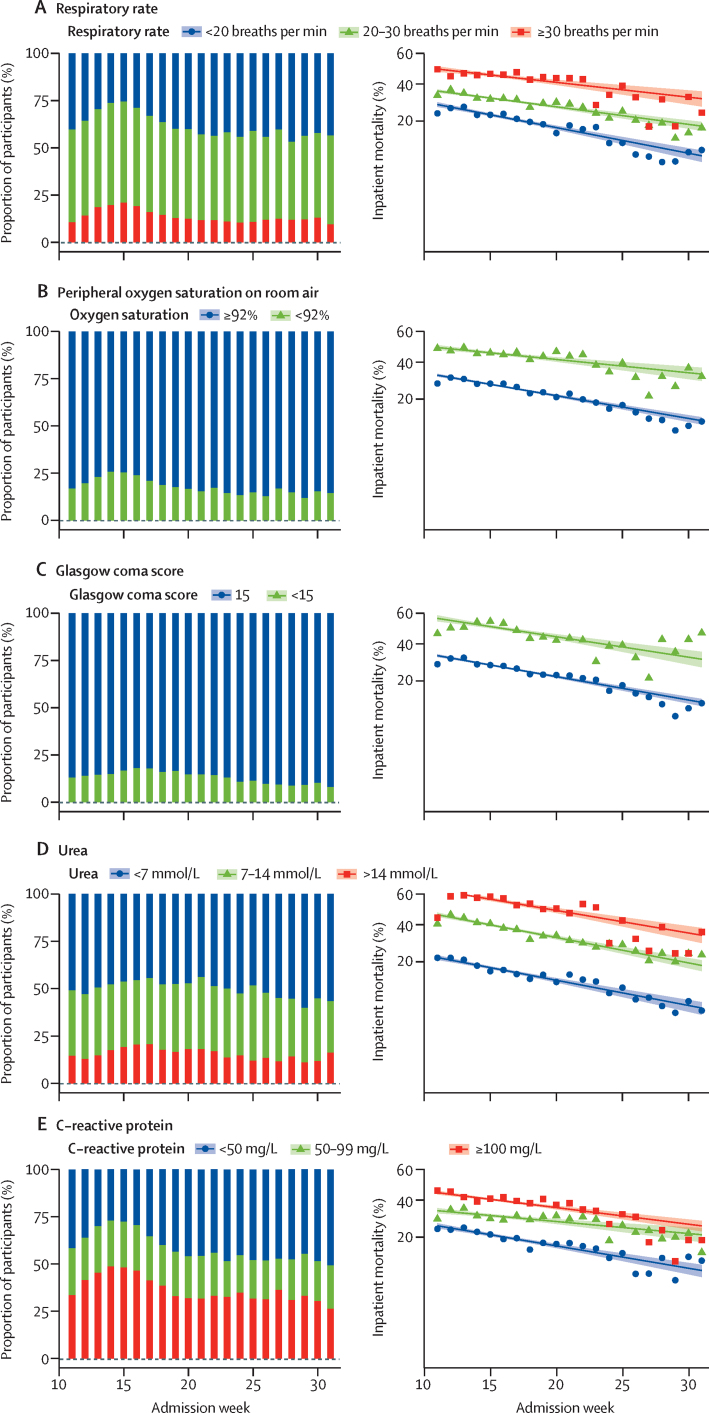
Proportion of adults admitted to hospital with COVID-19 and inpatient mortality between March 9 and Aug 2, 2020, stratified by respiratory rate (A), peripheral oxygen saturation on room air (B), Glasgow coma score (C), urea concentration (D), and C-reactive protein concentration (E) In A–E, all plots on the left show the proportion of adults admitted to hospital with COVID-19 by severity of illness at admission, and all plots on the right show the unadjusted in-hospital mortality rate each week during the time period. In all plots on the left, the proportions of participants are stacked by severity of illness at admission. Missing data are excluded from the figure.

Illness severity peaked from around March 30 to April 12, 2020 (weeks 14–16), when patients had faster respiratory rates, lower peripheral oxygen saturations on room air, and lower Glasgow coma scores, higher levels of acute kidney injury, and higher levels of inflammation at presentation to hospital than did patients admitted subsequently (figure 3). Patients presented later in their disease course at the beginning of the first wave compared with at the end of the first wave (median 4 days (IQR 0–7) in time period 1 *vs* 2 days (0–7) in time period 2 *vs* 3 days (0–7) in time period 3; table).

At the peak of admissions in time period 1, 38 139 (80·4%) patients admitted to hospital received supplementary oxygen. The proportion of patients receiving supplementary oxygen reduced consistently over subsequent weeks to approximately 50% in patients admitted from July onwards (table).

Most patients (54 632 [85·4%) of 63 972) admitted during the first wave were managed on the ward, with the proportion of patients admitted to critical care units peaking at the start of the first wave (7732 [16·3%] of 47 453 patients in time period 1; table; appendix pp 8,28). Compared with patients on wards, those in critical care units were younger (appendix pp 8–9) and more likely to be male (6433 [68·9%] of 9340 in critical care units *vs* 29 690 [54·3%] of 54  632 on wards; appendix pp 8–11). Patients with multiple comorbidities accounted for a substantial proportion of the total number of patients admitted (3733 [40·0%] of 9340 patients admitted to critical care units had two or more comorbidities *vs* 32 531 [59·5%] of 54 632 patients admitted to wards had two or more comorbidities; appendix pp 8–11). In critical care units, the proportions of younger patients and those with multiple comorbidities increased over time; a pattern that was also observed in patients admitted to wards (appendix pp 8–11).

The level of respiratory support received reduced over time in patients admitted to critical care units and wards (appendix pp 8–11). In critical care units, the requirement for invasive mechanical ventilation declined over time (4958 [64·1%] of 7732 patients in time period 1 *vs* 98 [29·4%] of 333 patients in time period 3), the proportion of patients requiring non-invasive ventilation increased substantially from 1768 (22·9%) patients in time period 1 to 157 (47·1%) patients in time period 3 (appendix pp 8–9). By comparison, the proportion of patients admitted to wards on non-invasive ventilation remained low, decreasing from 3390 (8·5%) of 39 721 patients in time period 1 to 115 (4·7%) of 2442 patients in time period 3 (appendix pp 10–11). By the end of the first wave, 42·7% of patients (1184 of 2775) admitted to hospital received no respiratory support (1184 [48·5%] of 2442 patients admitted to wards received no respiratory support; table; appendix pp 10–11). More information on the characteristics of patients admitted to critical care units and wards, and the proportions of patients receiving respiratory support are included in the appendix (pp 8–23).

The proportion of patients who received steroids increased from 7354 (15·5%) of 47 453 patients (2069 [26·8%] of 7732 patients in critical care units) in time period 1 to 910 (32·8%) of 2775 patients (223 [67·0%] of 333 patients in critical care units) in time period 3, mainly in patients receiving respiratory support (table; appendix pp 8–23, 29).

Unadjusted weekly in-hospital mortality at 28 days declined from 32·3% (95% CI 31·8–32·7) in the period from March 9 to April 26, 2020, to 16·4% (15·0–17·8) in the period from June 15 to August 2, 2020 (figure 1B; appendix p 24). This reduction in weekly in-hospital mortality at 28 days did not differ substantially in the sensitivity analyses, in which patients without an outcome were reclassified as survivors (appendix p 31), nor when patients who died in hospital more than 28 days after admission were included (a further 5% of patients died after 28 days; see appendix p 32 for subgroup analysis).

In-hospital mortality was higher with increasing age, increasing number of comorbidities, and male sex (figure 2). Over the course of the first wave, in-hospital mortality declined for all demographic categories, most notably in older patients (7867 [48·5%] of 16 233 patients aged ≥80 years died in time period 1 *vs* 239 [24·8%] of 965 patients aged ≥80 years in time period 3) and comorbid populations (figure 2; appendix p 7). Markers of increased severity of illness at presentation to hospital were associated with increased in-hospital mortality. In-hospital mortality declined for all markers of severity of illness over time (figure 3) and for patients treated on wards and in critical care units (appendix pp 24, 28, 29).

**Figure 4:**
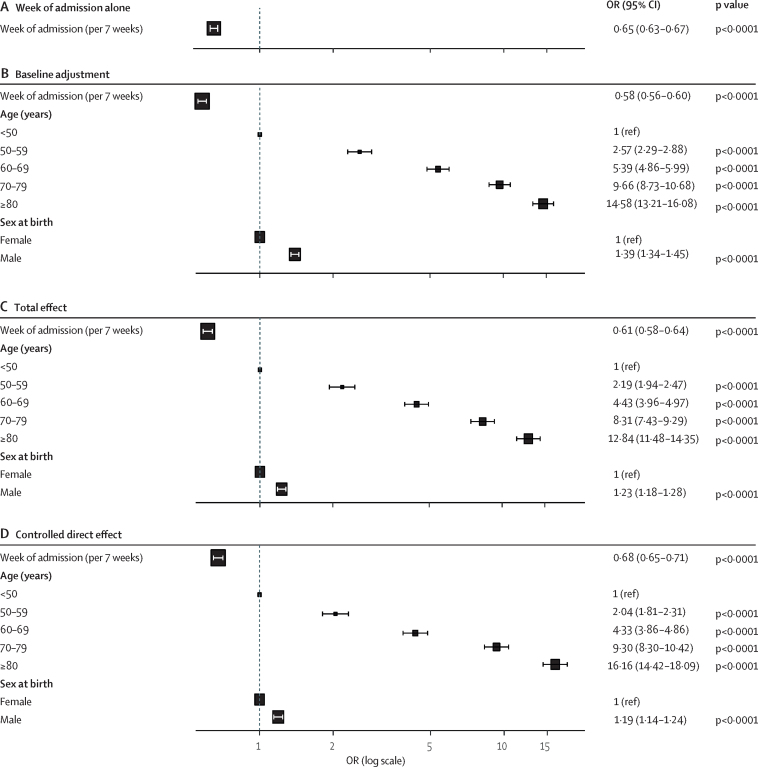
OR for in-hospital mortality for week of admission per 7-week period In-hospital mortality unadjusted for week of admission (A); adjusted for age, sex, deprivation, and hospital (B); adjusted for age, sex, deprivation quintile, severity of illness (respiratory rate, oxygen saturations, Glasgow coma score, serum urea concentration, and C-reactive protein), and number of comorbidities (C); and adjusted for age, sex, deprivation quintile, severity of illness, number of comorbidities, and potential mediators (maximal level of care, respiratory support, and treatment with steroids). Error bars represent 95% CIs. OR=odds ratio.

**Figure 5:**
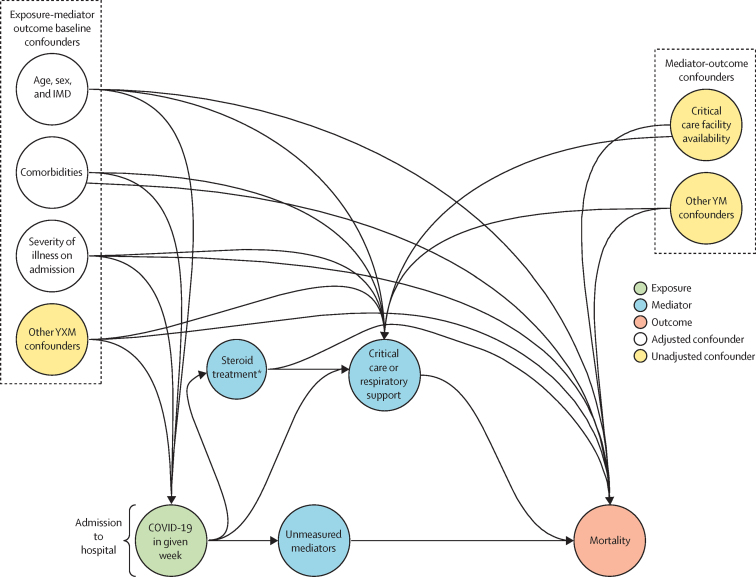
Causal graph with natural effects models mediation analysis The OR for the total natural indirect effect was 0·95 (95% CI 0·94–0·95, p<0·0001; percentage of effect through indirect path 22·2%; joint mediators) and for the pure natural direct effect was 0·84 (0·82–0·87, p<0·0001; percentage of effect from direct path 77·8%). IMD=index of multiple deprivation. YXM and YM=unmeasured confounders. OR=odds ratio. *Arrows from steroid treatment mediator confounders are not shown for clarity.

There was a 35% reduction in the odds of in-hospital mortality per 7-week time period (odds ratio [OR] 0·65 [95% CI 0·63–0·67], p<0·0001; figure 4). After adjustment for age, sex, deprivation, and hospital, the odds of in-hospital mortality per 7-week time period was 0·58 (0·56–0·60; p<0·0001). After additional adjustment for illness severity and number of comorbidities, the effect of week of admission on in-hospital mortality was similar to the effect before we adjusted for illness severity and comorbidity count (OR 0·61 [0·58–0·64], p<0·0001). With the addition of the mediator variables (respiratory support or steroid treatment), the OR reduced to 0·68 (0·65–0·71; p<0·0001). Case mix and illness severity accounted for 10·2% of the reduction in mortality, and 22·2% (OR 0·95 [0·94–0·95], p<0·0001) of the effect of week of admission on in-hospital mortality was mediated through respiratory care and steroid treatment (figure 5). There was a significant interaction between respiratory support and week of admission (p<0·0001).

There were substantial reductions in unadjusted in-hospital mortality between time period 1 and time period 3 in patients receiving no respiratory support (1339 [14·0%] of 9564 patients in time period 1 *vs* 71 [6·0%] of 1183 patients in time period 3), oxygen only (9496 [35·1%] of 27 054 patients admitted to wards in time period 1 *vs* 243 [21·3%] of 1141 patients admitted to wards in time period 3; and 228 [22·7%] of 1004 patients admitted to critical care units in time period 1 *vs* ten [12·8%] of 78 patients admitted to critical care units in time period 3), and non-invasive ventilation in critical care units (587 [33·2%] of 1768 patients in time period 1 *vs* 39 [24·8%] of 157 patients in time period 3) across all age groups (appendix pp 8–23). However, in-hospital mortality remained persistently high for patients receiving invasive mechanical ventilation (2034 [41·0%] of 4961 patients in time period 1 *vs* 41 [41·8%] of 98 patients in time period 3) and non-invasive ventilation on the ward (1626 [48·0%] of 3387 patients *vs* 51 [44·3%] of 115 patients). These differential changes in in-hospital mortality persisted after adjustment for demographic and severity of illness variables (appendix p 30).

## Discussion

Overall in-hospital mortality within 28 days of admission substantially decreased during the first wave of the pandemic in the UK. At the peak of admissions in late March and early April, 2020, patients were substantially more unwell at presentation to hospital and presented later from their onset of symptoms than those presenting to hospital at later months. There was a reduction in the level of respiratory support received; use of invasive ventilation reduced over time, and the proportion of patients receiving non-invasive ventilation increased. By late June and early August, almost half of patients admitted required no supplementary oxygen. The reduction in in-hospital mortality during the first wave was observed across all demographic groups, and was not fully accounted for by changes in the case-mix or a reduction in illness severity at hospital admission. One-fifth of the reduction in in-hospital mortality could be accounted for by changes in treatment, including respiratory care and steroid treatment.

ISARIC4C has recruited patients from hospitals across the UK, accounting for approximately two-thirds of patients admitted to hospital in the UK with COVID-19 in the first wave. Data were collected from arrival in the Emergency Department to discharge for patients managed both on wards and in critical care units, enabling us to review admission and mortality rates in whole hospitals rather than just in critical care units.

We showed a reduction in in-hospital mortality during the first wave that cannot be fully explained by baseline patient demographics or measured presenting severity of illness markers. These trends are consistent with those observed in New York hospitals,^20^ where mortality also significantly and progressively declined between March and August, 2020. Mortality rates in critical care units in the UK have also reduced over this time.^21^, ^22^ Most patients admitted to hospital during the first wave were older (ie, median age 70 years), had two or more comorbidities, and were of White ethnicity, and in-hospital mortality was highest in these groups. The case-mix has changed over the course of the pandemic, with an increase in the proportion of patients younger than 50 years and female patients who, both in our study and in other studies, have lower mortality rates than older groups and male patients.^23^, ^24^ However the declines in mortality were observed in all age groups, ethnic groups, and in both sexes. Shielding of susceptible groups was formally introduced in the UK on March 23, 2020,^25^ and patients presenting before shielding was introduced might have been more susceptible but not identifiable in our dataset. Severity of illness at presentation to hospital decreased, and the proportion of patients requiring no respiratory support increased at the end compared with at the start of the first wave. SARS-CoV-2 is transmitted predominantly by respiratory droplets; therefore, physical distancing, lockdown (between March 23, 2020, and July 4, 2020),^26^ and widespread adoption of face masks could have reduced viral load (ie, the infectious dose) at the point of transmission,^27^ thus reducing severity of illness in infected patients.^28^ Compared with March and April, 2020, patients presented earlier in the disease course in June and July, 2020, and length of stay of non-survivors in critical care units increased, consistent with patients presenting earlier in their illness and fewer in extremis. Our data do not include community factors; however, it is possible that changes in health-seeking behaviour might have led to patients attending hospitals more easily and earlier.

Hospital admissions in the UK peaked at approximately 3000 patients admitted per day in early April, 2020, with the ICU caseload peaking shortly after this point^1^, ^2^ before gradually declining to a plateau of approximately 100 patients admitted per day by July, 2020.^1^ The UK has a small health-care workforce, with few hospital and critical care beds per capita relative to other high income countries.^29^, ^30^ At the start of the pandemic, the UK had high occupancy of hospital beds and little spare capacity.^29^ However, during the rapid response to COVID-19, an additional 2711 beds compatible with mechanical ventilation across England were made available, reflecting a 53% increase in capacity.^31^ National reported occupancy in critical care units never exceeded 62%, although local peaks in critical care unit occupancy were much higher than this value, with 52 hospitals across England reaching 100% occupancy at one or more points during the first wave of the pandemic.^30^, ^31^ The effect of increased patient numbers could have been different depending on the type and size of the hospital, with smaller hospitals reaching full capacity sooner than larger hospitals. Even before the pandemic, critical care capacity strain was associated with increased mortality.^32^ The rapid increase in critical care beds required redeployment of non-critical care staff, and in some UK regions, increased nursing staff-to-patient ratios, which could have influenced early patient outcomes.^33^

In the first time period late March, early April, we found that a higher proportion of patients were admitted to critical care units than later. Patients in critical care units at this time were considerably younger than those on wards; even so, in-hospital mortality was much higher for COVID-19 than for other severe acute respiratory infections, such as viral pneumonia.^7^ During the peak admission period, the proportion of patients aged older than 80 years and the proportion of patients with two or more comorbidities admitted to critical care units was lower than after this time point; however, this pattern reflected the demographic of patients admitted to wards during the peak admission period.

A fifth of the reduction in in-hospital mortality in our study can be explained by changes in respiratory support and steroid treatment, together with associated changes in clinical decision making associated with respiratory support. At the beginning of the pandemic, much attention was given to early intubation and to different ventilator management practices based on different presumed phenotypes. These practices changed as clinicians started to manage acute respiratory distress syndrome (ARDS) from COVID-19 as routine ARDS. The proportion of patients receiving invasive ventilation reduced over time; however, in-mortality remained persistently high after adjusting for patient demographics and illness severity, as shown in our study. It would be unwise to interpret this association as causal, as use of invasive mechanical ventilation is reserved for patients with the most severe illness, and there was an overall reduction in mortality in patients with COVID-19 admitted to critical care units in our study. There are several potential explanations for this finding. First, the change in case-mix might not have been adequately captured by multimorbidity and age; a higher proportion of older patients (ie, those older than 70 years) and patients with comorbidities were ventilated later in the first wave, potentially at a time when there was more critical care capacity than at the start of the first wave. Second, critical care practices changed, with increasing use of non-invasive ventilation over time, and only patients presenting in extremis or those who did not respond to a trial of non-invasive ventilation received invasive mechanical ventilation. This change in practice could be partly due to the changing case-mix, but also due to increasing clinician familiarity with the use of non-invasive ventilation, and an improving ability to identify which patients might benefit from this intervention.^34^ It is possible that patients admitted early in the first wave who received invasive mechanical ventilation would have received non-invasive ventilation if they had been admitted later in the first wave, and that they would have survived regardless of the mode of ventilation. Therefore, compared with patients who received invasive mechanical ventilation early in the first wave, those admitted later who received invasive mechanical ventilation were a more severely ill population who had failed to respond to treatments and would die if not offered invasive mechanical ventilation. This notion is supported by the significant increase in the effect of respiratory care over time; allocation to respiratory support was linked to better prediction of outcome by clinicians over time in our study. Ongoing trials comparing the use of non-invasive ventilation and invasive mechanical ventilation in critically ill patients with COVID-19 will help to overcome this selection bias and confounding by indication to ascertain whether patient selection or non-invasive ventilation itself is improving outcomes in critically ill patients with COVID-19.

In our study, in-hospital mortality rates in patients receiving non-invasive ventilation on the ward were higher than in patients receiving oxygen and non-invasive ventilation being managed in critical care units. Together with a higher proportion of patients with comorbidities, particularly dementia and chronic pulmonary and cardiac disease, this observation could indicate that the benefit of these treatments in this group of patients is limited, and could also indicate a potential ceiling of treatment for patients receiving non-invasive ventilation on the ward. In-hospital mortality was high in older patients (ie, those older than 80 years) who received invasive ventilation. The benefit of ICU admission for older frail patients remains uncertain, as rates of mortality and long-term functional impairment in survivors are high in this population.^35^ Critical care interventions might not be associated with improved outcomes in this group. In a previous study, protocolised ICU referral in patients aged 75 years and older led to significantly higher ICU admission rates but had no significant effect on mortality, functional status, or health-related quality of life.^36^ It is essential that meaningful discussions about the available treatment options, as well as the risks and benefits of each, are discussed with these patients and their families.^37^ Such discussions should also emphasise that much of the potential benefit of care can be derived without the need for ICU level care.

Clinical practice outside critical care has also changed during the first wave, with increasing clinical familiarity with COVID-19. Clinicians might have become more alert to deterioration, which can occur rapidly during COVID-19 infection, and might not have been accurately captured by our data collection. Corticosteroid treatment^9^, ^10^ substantially benefits subgroups of hospital inpatients, and trials of other treatments, including anticoagulants, anti-inflammatory drugs, anti-viral drugs, convalescent plasma, and non-invasive ventilation are ongoing.^9^, ^10^ These evidence-based COVID-19 interventions highlight the critical importance of suitably powered randomised controlled trials for drug evaluation, even in outbreak situations.

This observational cohort study has some limitations. We did not record treatment escalation plans, but we were able to examine changing case-mixes in the ward and in critical care units. Due to the nature of the pandemic, there were more missing data than would normally be expected in a prospective cohort study, but missing data were handled with the appropriate methods, as discussed in the methods and appendix. We were unable to comment on community factors leading up to admission, and indeed, for patients who were not admitted to hospital. The change in management of ventilated patients would have benefitted from measurement of physiological variables; however, we did not collect this information. We also did not include unproven treatments being investigated in randomised controlled trials, which could have had either a beneficial or harmful effect. We were unable to locate any publicly available data for strain in the National Health Service (NHS) during the first wave (ie, excessive demand on the strength, resources, or abilities of hospitals). Although data on the proportion of ICUs that had reached so-called surge capacity were available for Scotland, equivalent data for England were not available. Furthermore, different hospitals had surges in both daily admissions and number of in-patients at different times; therefore, defining a time when hospitals were working beyond their pre-COVID-19 capacity was complex. We were unable to identify individual hospital strain; however, we did include hospital as a variable in our model. We used components of the ISARIC4C Mortality Score for severity of illness at presentation. This is a pragmatic score-based model, therefore discrimination and calibration might be reduced in subgroups of patients. Furthermore, with changes in treatment and outcomes over time, predicted in-mortality could be overestimated. However, in internal temporal and external validation studies, performance of this model has remained good. As this was an observational study, we were unable to assign causality, and unmeasured confounding might remain. The issue of selection bias for those admitted to critical care, compared with those who are cared for on the ward (ie, collider bias), is well established in critical care epidemiology literature, and its effect on associations is understood. The comparison of ward versus critical care cohorts is absent from most other studies.

In conclusion, in-hospital mortality rates in patients with COVID-19 declined in the UK during the first wave of the pandemic. This reduction persisted after adjusting for illness severity and changes in patient case-mix. Patients were most severely unwell at hospital presentation at the start of the first wave and presented later in the disease course than did patients at the end of the first wave. A significant proportion of the reduction in in-hospital mortality can be explained by changes in clinical management, including respiratory support and steroid treatment. Hospital practice has changed; the use of non-invasive ventilation has increased substantially, and many patients have been included in drug trials and trials of other treatments, which might explain the reduction in in-hospital mortality and inform future waves. In-hospital mortality remained high for patients receiving invasive ventilation and non-invasive ventilation on the ward, and these populations should be a priority in ongoing research.

## Data sharing

The COVID-19 Clinical Information Network (CO-CIN) data were collated by ISARIC4C Investigators. The application process for access to the data is available on the ISARIC4C website.

## Declaration of interests

ABD reports grants from Department of Health and Social Care (DHSC), during the conduct of the study; and grants from Wellcome Trust, outside of the submitted work. LT reports grants from Wellcome Trust. JSNV-T reports salary support from DHSC, during the conduct of the study, and is seconded to DHSC. PJMO reports personal fees from consultancies and from the European Respiratory Society; grants from the Medical Research Council (MRC), MRC Global Challenge Research Fund, EU, NIHR BRC, MRC/GSK, Wellcome Trust, NIHR (Health Protection Research Unit [HPRU] in Respiratory Infection); and is an NIHR senior investigator outside of the submitted work; his role as President of the British Society for Immunology was unpaid but travel and accommodation at some meetings was provided by the Society. JKB reports grants from MRC UK. MGS reports grants from DHSC NIHR UK, MRC UK, and HPRU in Emerging and Zoonotic Infections, University of Liverpool during the conduct of the study; and other from Integrum Scientific (Greensboro, NC, USA) outside of the submitted work. RHM reports grants from BREATHE, the health data research hub for respiratory health [MC_PC_19004]. BREATHE is funded through the UK Research and Innovation Industrial Strategy Challenge Fund and is delivered by Health Data Research UK. All other authors declare no competing interests.
