# Supplementary material for: Changes in in-hospital mortality in the first wave of COVID-19: a multicentre prospective observational cohort study using the WHO Clinical Characterisation Protocol UK
Source: Lancet Respir Med. 2021 Jul;9(7):773–85. doi: 10.1016/S2213-2600(21)00175-2 (PMC8121531; doi:10.1016/S2213-2600(21)00175-2)
Supplement: Supplementary appendix [file mmc1.pdf]

THE LANCET
Respiratory Medicine

Supplementary appendix

This appendix formed part of the original submission and has been peer reviewed. We post it as supplied by the authors.

Supplement to: Docherty AB, Mulholland RH, Lone NI, et al. Changes in in-hospital mortality in the first wave of COVID-19: a multicentre prospective observational cohort study using the WHO Clinical Characterisation Protocol UK. *Lancet Respir Med* 2021; published online May 14. http://dx.doi.org/10.1016/S2213-2600(21)00175-2.

Supplementary information: Changes in UK hospital mortality in the first wave of COVID-19: the ISARIC WHO Clinical Characterisation Protocol prospective multicentre observational cohort study.

Annemarie B Docherty*1, Rachel H Mulholland*2, Nazir I Lone3, Christopher P Cheyne4, Daniela De Angelis5, Karla Diaz-Ordaz6, Cara Donegan7, Thomas M Drake1, Jake Dunning9, Sebastian Funk6, Marta García-Fiñana4, Michelle Girvan8, Hayley E Hardwick7, Janet Harrison8, Antonia Ho10, David M Hughes4, Ruth H Keogh6, Peter D Kirwan5, Gary Leeming4, Jonathan S Nguyen-Van-Tam11, Riinu Pius1, Clark D Russell12, Rebecca Spencer7, Brian DM Tom5, Lance Turtle7, Peter JM Openshaw9, J Kenneth Baillie13, Ewen M Harrison**1 Malcolm G Semple**7,14, for ISARIC4C investigators

Appendix 1

Definitions

Term	Definition
Acute hospitals	Provides general short-term healthcare treatment such as treatment for severe injury, period of illness, urgent medical condition, or to recover from surgery. In the NHS, it often includes services such as accident and emergency (A&E) departments, inpatient and outpatient medicine and surgery
Non-invasive ventilation (NIV)	bilevel and continuous positive airway pressure
Invasive mechanical ventilation (IMV)	Positive pressure ventilation delivered via a tube inserted into the trachea (through the mouth, nose, or skin in the neck)
Level of care	"Ward": Level 0/1<br>- Ward based care where the patient does not require organ support (for example, they may need an IV, or oxygen by face mask)<br>Critical Care: Level 2 or 3<br>- Level 2: High dependency unit (HDU). Patients needing single organ support (excluding mechanical ventilation) such as renal haemofiltration or ionotropes and invasive BP monitoring. They are staffed with one nurse to two patients<br>- Level 3: Intensive care (ICU). Patients requiring two or more organ support (or needing mechanical ventilation alone). Staffed with one nurse per patient and usually with a doctor present in the unit 24 hours per day.

Time-line of UK response to COVID-19

Date	Event
31/01/2020	1st cases in the UK
10/02/2020	SARS-CoV-2 serious and imminent threat to public health in UK
21/02/2020	COVID-19: guidance for ambulance services (https://www.gov.uk/government/publications/covid-19-guidance-for-ambulance-trusts/covid-19-guidance-for-ambulance-trusts#contents)
05/03/2020	First UK COVID-19 death reported
12/03/2020	National strategy changed from containment to admission based on clinical need
17/03/2020	NICE Specialty guides, including Accident and Emergency (https://www.nice.org.uk/covid-19/specialty-guides)
17/03/2020	Actions to free up inpatient and critical care capacity (postpone non-urgent elective operations, discharge inpatients medically fit to leave, block buying capacity in independent providers) https://www.england.nhs.uk/coronavirus/wp-content/uploads/sites/52/2020/03/20200317-NHS-COVID-letter-FINAL.pdf
20/03/2020	NICE guideline: critical care in adults (NG159)
21/03/2020	Shielding of vulnerable people
23/03/2020	UK lockdown
15/04/2020	NHS advice against high flow nasal oxygen (aerosolisation, hospital oxygen supplies) https://www.bmj.com/content/369/bmj.m2446
17/04/2020	Government extends access to testing for those with symptoms of COVID-19 across England to additional front line workers and members of their household https://www.gov.uk/government/news/government-to-extend-testing-for-coronavirus-to-more-frontline-workers
26/05/2020	Remdesivir available for strict clinical criteria through Early Access to Medicines Scheme (EAMS) https://www.cas.mhra.gov.uk/ViewandAcknowledgment/ViewAttachment.aspx?Attachment_id=103580
05/06/2020	RECOVERY trial: no clinical benefit from use of hydroxychloroquine in hospitalised patients with covid-19
08/06/2020	RECOVERY trial press conference: Dexamethasone shown to reduce mortality in hospital subpopulation
15/06/2020	All hospital staff should wear a face mask https://www.gov.uk/government/publications/wuhan-novel-coronavirus-infection-prevention-and-control/new-government-recommendations-for-england-nhs-hospital-trusts-and-private-hospital-providers
04/07/2020	UK lockdown eased

Setting

Participation by every hospital was mandated by the Chief Medical Officer in 2012, and re-emphasised at the beginning of the pandemic. Over 2,500 research nurses, clinicians and medical students contributed to the collection of the data, which represented approximately two thirds of all patients admitted to all hospitals in England, Scotland and Wales. Due to overwhelming numbers, and secondment of research staff back to clinical practice, many sites were overwhelmed and unable to maintain data collection for all patients.

Exclusions

For this analysis, we included only acute general hospitals. Community hospitals providing long-term treatment and residential mental health hospitals were excluded since patient populations were very different and were therefore not comparable. We excluded 'nosocomial' infection as patients with onset of COVID-19 symptoms more than 5 days after they were admitted to hospital for a separate condition.[1] We excluded patients where data entries for outcome date and age were clearly erroneous. Onset of symptoms to admission ≥21 days were assumed to be errors.

Site training emphasised that only patients who tested positive (using reverse transcriptase polymerase chain reaction, RT-PCR) for COVID-19 were eligible for enrolment. However a significant proportion of patients had missing PCR results. A significant proportion of patients, particularly early on in the first wave, had clinical symptoms and signs consistent with COVID-19, yet either a negative test. Furthermore, access to testing was limited at the beginning of the first wave. We have included patients with both positive PCR, and also high clinical suspicion of covid-19 without the supporting PCR test.

Variables

Age: We cut age into 4 categories - <50yrs, 50-69yrs, 70-79yrs, 80yrs+. These age categories are consistent with our other published studies. The age cut-points are based on the S shape curve of age vs mortality (flat to 50, flat after 80), and we have divided the middle gradient into 2 using the methods of Barrio et al[2] to identify cut points on the basis of slopes and clinical significance. Our methods are explained and referenced in the 4C Risk score supplementary material.[3]

Comorbidities: asthma, diabetes, chronic cardiac disease (excluding hypertension), chronic haematologic disease, chronic kidney disease, chronic neurological disorder, chronic pulmonary disease (excluding asthma), dementia, HIV/AIDS, malignancy, malnutrition, mild to severe liver disease, clinician-assigned obesity and rheumatologic disorder.

Missing data: Missing data patterns were analysed (finalfit package) and data were considered missing at random (as opposed to missing completely at random). Multiple imputation of missing values was performed (mice package) with 10 iterations to create 10 imputed sets using the 35 explanatory variables plus outcome. Imputation methods were continuous variables: predictive mean matching; 2-level factors: binary logistic regression; and >2-level factors: polytomous regression (all considered unordered). Distributions of imputed variables were inspected across iterations.

High Flow Nasal Oxygen (HFNO): Due to differing interpretation of the high flow variable by staff uploading the data during the pandemic, we were unable to differentiate between HFNO and patients on standard oxygen masks. NHS England advised against HFNC in the first wave due to concerns regarding aerosolisation and hospital oxygen supplies (https://www.hee.nhs.uk/coronavirus-covid-19/coronavirus-covid-19-overview), although it has subsequently been used increasingly in the second wave. We believe the majority of patients classified as high flow will have been on standard oxygen masks, and we have categorised it accordingly.

Pharmaceutical interventions: Only corticosteroids have had a proven mortality benefit during the first wave in the UK. Remdesivir has been shown to shorten hospital stay

(https://www.nejm.org/doi/full/10.1056/NEJMoa2007764) but had no significant reduction in mortality. As our primary endpoint in this study was mortality, we chose not to explore this further. In the UK, the MHRA and Scottish government felt that the benefit for remdesivir in critically ill patients was uncertain, and it was approved for use on 26th May 2020 through an interim clinical commissioning policy with strict clinical criteria https://www.gov.scot/publications/coronavirus-covid-19-guidance-on-critical-care-management-of-adult-patients/pages/clinical-management-of-patients-with-covid-19-infection/#Remdesivir. Very few patients received remdesivir in the UK outside clinical trials in the duration of this analysis.

Statistical methods

Secondary outcome: A Bayesian approach was taken to facilitate the use of imputed datasets with GAMs, and to allow the easy determination of probabilities of interest. Continuous and binary data were centred and normalised. Weakly informative priors were used for regression coefficients (Student's t-distribution, df = 7, location = 0, scale = 2.5) and for the standard deviation of smooth terms (exponential, rate = 1). Each imputed data set (n = 10) was fitted separately (chains = 4, total iterations = 5000, warm-up = 1000, random initiation values from -2 to 2) and results pooled across models. Posterior distributions were sampled from pooled data. Convergence was ensured by inspecting chain trace, density, and autoregression plots, and determining the Gelman/Rubin potential scale reduction factor (R-hat).

Models incorporated variables previously shown to be associated with in-hospital mortality: age, sex, comorbidity count, and severity of illness (RR, SpO2, GCS, urea, CRP)). Based on our previous work, a three-way interaction was included between admission week, age, and maximum level of respiratory support. Multivariable GLMs were used to fit in-hospital mortality to week of admission (straight lines on log odds scale). Multivariable GAMs using restricted cubic splines were then used to allow the response to vary for all continuous variables. Admission week and age smooth terms were allowed to vary by maximum respiratory support and a tensor product smooth term used to model the admission week and age interaction. Unidentified residual confounding was represented by "other YM confounders" and "other YXM confounders" (Figure E1)

Statistical disclosure control (SDC) measures were adopted in the summary statistics tables to reduce the risk of patient confidentiality. If cell was <5 then it was either removed (and replaced with NA) or aggregated with another level to hide small numbers. Note NA could be suppressed or 0. If small cell was an 'Unknown' category these were left as we are assuming they are random by chance.

Appendix 2: Baseline characteristics tables

Tables

Table E1: Baseline characteristics comparing PCR positive vs negative vs missing patients

Type	Characteristic	PCR Result N (%) or Mean (SD)			
		Positive	Negative	No test recorded	Missing
	**Total**	40449 (63.2)	1486 (2.3)	3309 (5.2)	18728 (29.3)
**Outcome**	**Mortality within 28 days**	12855 (31.8)	287 (19.3)	1012 (30.6)	5027 (26.8)
**Patient characteristics**	**Country**				
	England	37535 (92.8)	1402 (94.3)	3200 (96.7)	16357 (87.3)
	Scotland	1948 (4.8)	60 (4.0)	86 (2.6)	613 (3.3)
	Wales	966 (2.4)	24 (1.6)	23 (0.7)	1758 (9.4)
	**Age (grouped)**				
	<50	5258 (13.0)	239 (16.1)	452 (13.7)	2461 (13.1)
	50-69	11878 (29.4)	499 (33.6)	935 (28.3)	4967 (26.5)
	70-79	9015 (22.3)	340 (22.9)	751 (22.7)	4174 (22.3)
	80+	14298 (35.3)	408 (27.5)	1171 (35.4)	7126 (38.0)
	**Age (continuous)**				
	Mean (SD)	70.4 (16.8)	67.6 (17.2)	70.4 (16.9)	71.2 (17.3)
	**Sex**				
	Female	17093 (42.3)	662 (44.5)	1412 (42.7)	8682 (46.4)
	Male	23356 (57.7)	824 (55.5)	1897 (57.3)	10046 (53.6)
	**Ethnic group**				
	White	30002 (74.2)	1199 (80.7)	2315 (70.0)	13231 (70.6)
	South Asian	1774 (4.4)	70 (4.7)	289 (8.7)	972 (5.2)
	East Asian	301 (0.7)	12 (0.8)	21 (0.6)	117 (0.6)
	Black	1550 (3.8)	45 (3.0)	148 (4.5)	549 (2.9)
	Other Ethnic Minority	2798 (6.9)	80 (5.4)	235 (7.1)	1137 (6.1)
	Missing	4024 (9.9)	80 (5.4)	301 (9.1)	2722 (14.5)
	**Number of comorbidities**				
	0	8940 (22.1)	308 (20.7)	1113 (33.6)	2949 (15.7)
	1	9880 (24.4)	354 (23.8)	617 (18.6)	3547 (18.9)
	2+	21629 (53.5)	824 (55.5)	1579 (47.7)	12232 (65.3)
	**Health worker**	2103 (5.2)	74 (5.0)	170 (5.1)	887 (4.7)
**Severity of illness**	**Asymptomatic**	938 (2.3)	33 (2.2)	142 (4.3)	2716 (14.5)
	**Symptom onset (days)***				
	Mean (SD)	4.8 (5.1)	4.9 (5.6)	5.1 (5.3)	4.6 (5.2)
	**Length of stay (days)**				
	Mean (SD)	11.5 (11.6)	9.6 (11.1)	11.3 (13.4)	12.9 (13.7)
	**ISARIC 4C Score**				
	Low (0-3)	1816 (4.5)	106 (7.1)	140 (4.2)	916 (4.9)
	Intermediate (4-8)	6425 (15.9)	272 (18.3)	416 (12.6)	2882 (15.4)
	High (9-14)	14180 (35.1)	606 (40.8)	947 (28.6)	7234 (38.6)
	Very high (15+)	4133 (10.2)	134 (9.0)	300 (9.1)	1965 (10.5)
	Missing	13895 (34.4)	368 (24.8)	1506 (45.5)	5731 (30.6)
	**Respiratory rate (breaths/min)**				
	<20	11749 (29.0)	393 (26.4)	815 (24.6)	6543 (34.9)
	20-30	20435 (50.5)	768 (51.7)	1332 (40.3)	8685 (46.4)
	>=30	6846 (16.9)	278 (18.7)	524 (15.8)	2760 (14.7)
	Missing	1419 (3.5)	47 (3.2)	638 (19.3)	740 (4.0)
	**Peripheral oxygen saturation on room air (%)**				
	>=92	30038 (74.3)	1111 (74.8)	2041 (61.7)	14384 (76.8)
	<92	8728 (21.6)	326 (21.9)	617 (18.6)	3598 (19.2)
	Missing	1683 (4.2)	49 (3.3)	651 (19.7)	746 (4.0)
	**Glasgow coma score**				
	15	30803 (76.2)	1185 (79.7)	2023 (61.1)	14572 (77.8)
	<15	5596 (13.8)	192 (12.9)	420 (12.7)	2598 (13.9)
	Missing	4050 (10.0)	109 (7.3)	866 (26.2)	1558 (8.3)
	**Urea (mmol/L)**				
	<7	15117 (37.4)	676 (45.5)	1001 (30.3)	7232 (38.6)
	7-14	10963 (27.1)	407 (27.4)	691 (20.9)	5339 (28.5)

Type	Characteristic	PCR Result N (%) or Mean (SD)			
		Positive	Negative	No test recorded	Missing
	>14	5558 (13.7)	181 (12.2)	376 (11.4)	2612 (13.9)
	Missing	8811 (21.8)	222 (14.9)	1241 (37.5)	3545 (18.9)
	**C-reactive protein (mg/dL)**				
	<50	10630 (26.3)	526 (35.4)	757 (22.9)	5613 (30.0)
	50-99	7986 (19.7)	234 (15.7)	561 (17.0)	3458 (18.5)
	>=100	14865 (36.7)	543 (36.5)	955 (28.9)	6005 (32.1)
	Missing	6968 (17.2)	183 (12.3)	1036 (31.3)	3652 (19.5)
**Respiratory support and treatments**	**Level of care**				
	Critical care	6435 (15.9)	247 (16.6)	413 (12.5)	2245 (12.0)
	Ward	34014 (84.1)	1239 (83.4)	2896 (87.5)	16483 (88.0)
	**Respiratory support**				
	None	7718 (19.1)	366 (24.6)	1276 (38.6)	5918 (31.6)
	Oxygen only	24251 (60.0)	811 (54.6)	1501 (45.4)	9851 (52.6)
	Non-invasive	4445 (11.0)	196 (13.2)	270 (8.2)	1759 (9.4)
	Invasive	4035 (10.0)	113 (7.6)	262 (7.9)	1200 (6.4)
	**Steroids**				
	Yes	6319 (15.6)	305 (20.5)	425 (12.8)	3482 (18.6)
	No	31876 (78.8)	1123 (75.6)	2162 (65.3)	14607 (78.0)
	Missing	2254 (5.6)	58 (3.9)	722 (21.8)	639 (3.4)

Table E2: Individual comorbidities of patients presenting in the first wave, divided by 3 equal time points.

**Comorbidity**	**1 - Weeks 11 to 17** 9 Mar to 26 Apr 2020	**2 - Weeks 18 to 24** 27 Apr to 14 Jun 2020	**3 - Weeks 25 to 31** 15 Jun to 2 Aug 2020
**AIDS/HIV**	178 (0.4%)	41 (0.3%)	13 (0.5%)
**Asthma**	6373 (13.4%)	1612 (11.7%)	336 (12.1%)
**Chronic cardiac disease**	13837 (29.2%)	4745 (34.5%)	883 (31.8%)
**Chronic hematologic disease**	1833 (3.9%)	633 (4.6%)	118 (4.3%)
**Chronic kidney disease**	60 (15.7%)	2551 (18.6%)	483 (17.4%)
**Chronic neurological disorder**	5338 (11.2%)	1829 (13.3%)	304 (11%)
**Chronic pulmonary disease**	7773 (16.4%)	2482 (18.1%)	482 (17.4%)
**Dementia**	6992 (14.7%)	2666 (19.4%)	343 (12.4%)
**Diabetes Type**			
Type 1	720 (1.5%)	393 (2.9%)	66 (2.4%)
Type 2	6166 (13%)	3275 (23.8%)	659 (23.7%)
**Hypertension***	11061 (23.3%)	6133 (44.6%)	1185 (42.7%)
**Malignant neoplasm**	4167 (8.8%)	1495 (10.9%)	315 (11.4%)
**Malnutrition**	988 (2.1%)	375 (2.7%)	68 (2.5%)
**Mild to severe liver disease**	1342 (2.8%)	527 (3.8%)	104 (3.7%)
**Obesity**	4923 (10.4%)	1342 (9.8%)	294 (10.6%)
**Rheumatologic disorder**	4804 (10.1%)	1674 (12.2%)	308 (11.1%)
* Hypertension added May 2020			

Table E3: Baseline characteristics of ICU patients presenting in the first wave, divided by 3 equal time points (N=9,340). Disclosure threshold of 5 was used. Proning is for all patients who were proned, whether awake or intubated.

Type	Characteristic	1 - Weeks 11 to 17<br>9 Mar to 26 Apr 2020	2 - Weeks 18 to 24<br>27 Apr to 14 Jun 2020	3 - Weeks 25 to 31<br>15 Jun to 2 Aug 2020
	**Total**	7732	1275	333
Patient characteristics	**Country**			
	England	6973 (90.2%)	1153 (90.4%)	312 (93.7%)
	Scotland	460 (5.9%)	53 (4.2%)	NA
	Wales	299 (3.9%)	69 (5.4%)	21 (6.3%)
	**Age (grouped)**			
	<50	1564 (20.2%)	266 (20.9%)	79 (23.7%)
	50-69	4194 (54.2%)	637 (50%)	146 (43.8%)
	70-79	1499 (19.4%)	244 (19.1%)	69 (20.7%)
	80+	475 (6.1%)	128 (10%)	39 (11.7%)
	**Age (continuous)**			
	Median (IQR)	61 (IQR=17.9)	62 (IQR=20.4)	64 (IQR=22.4)
	Mean (SD)	60.6 (SD=13.4)	61.3 (SD=15)	61.7 (SD=16.1)
	**Sex**			
	Female	2314 (29.9%)	465 (36.5%)	128 (38.4%)
	Male	5418 (70.1%)	810 (63.5%)	205 (61.6%)
	**Ethnic group**			
	White	4754 (61.5%)	869 (68.2%)	209 (62.8%)
	South Asian	562 (7.3%)	87 (6.8%)	54 (16.2%)
	East Asian	124 (1.6%)	10 (0.8%)	NA
	Black	468 (6.1%)	57 (4.5%)	8 (2.4%)
	Other Ethnic Minority	831 (10.7%)	112 (8.8%)	40 (12%)
	Unknown	993 (12.8%)	140 (11%)	22 (6.6%)
	**Number of comorbidities**			
	0	2655 (34.3%)	259 (20.3%)	57 (17.1%)
	1	2238 (28.9%)	318 (24.9%)	80 (24%)
	2+	2839 (36.7%)	698 (54.7%)	196 (58.9%)
	**AIDS/HIV**	54 (0.7%)	9 (0.7%)	NA
	**Asthma**	1242 (16.1%)	187 (14.7%)	54 (16.2%)
	**Chronic cardiac disease**	1147 (14.8%)	250 (19.6%)	76 (22.8%)
	**Chronic hematologic disease**	222 (2.9%)	34 (2.7%)	10 (3%)
	**Chronic kidney disease**	72 (7.4%)	129 (10.1%)	35 (10.5%)
	**Chronic neurological disorder**	375 (4.8%)	87 (6.8%)	25 (7.5%)
	**Chronic pulmonary disease**	693 (9%)	172 (13.5%)	61 (18.3%)
	**Dementia**	141 (1.8%)	45 (3.5%)	5 (1.5%)
	**Diabetes Type**			
	Type 1	109 (1.4%)	49 (3.8%)	11 (3.3%)
	Type 2	954 (12.3%)	343 (26.9%)	78 (23.4%)
	**Hypertension**	1512 (19.6%)	522 (40.9%)	138 (41.4%)
	**Malignant neoplasm**	369 (4.8%)	101 (7.9%)	20 (6%)
	**Malnutrition**	71 (0.9%)	19 (1.5%)	NA
	**Mild to severe liver disease**	172 (2.2%)	45 (3.5%)	13 (3.9%)
	**Obesity**	1524 (19.7%)	282 (22.1%)	71 (21.3%)
	**Rheumatologic disorder**	531 (6.9%)	102 (8%)	21 (6.3%)
	**Health worker**	682 (8.8%)	177 (13.9%)	15 (4.5%)
Severity of illness	**Asymptomatic**	184 (2.4%)	91 (7.1%)	39 (11.7%)
	**Symptom onset (days)***			
	Median (IQR)	7 (IQR=7)	5 (IQR=7)	4 (IQR=6)
	Mean (SD)	7 (SD=4.9)	5.5 (SD=5.1)	4.9 (SD=4.6)
	**Length of stay (days)**			
	Median (IQR)	14 (IQR=18)	14 (IQR=17)	11 (IQR=13)
	Mean (SD)	19.7 (SD=17.5)	17.9 (SD=14.9)	13.8 (SD=10.1)
	**ISARIC 4C Score**			
	Low (0-3)	215 (2.8%)	62 (4.9%)	17 (5.1%)
	Intermediate (4-8)	1797 (23.2%)	324 (25.4%)	90 (27%)
	High (9-14)	2677 (34.6%)	483 (37.9%)	122 (36.6%)
	Very high (15+)	492 (6.4%)	89 (7%)	20 (6%)

	Unknown	2551 (33%)	317 (24.9%)	84 (25.2%)
	**Respiratory rate (breaths/min)**			
	<20	1317 (17%)	285 (22.4%)	68 (20.4%)
	20-30	3737 (48.3%)	640 (50.2%)	170 (51.1%)
	>=30	2379 (30.8%)	315 (24.7%)	89 (26.7%)
	Unknown	299 (3.9%)	35 (2.7%)	6 (1.8%)
	**Peripheral oxygen saturation on room air (%)**			
	>=92	4552 (58.9%)	841 (66%)	221 (66.4%)
	<92	2918 (37.7%)	408 (32%)	105 (31.5%)
	Unknown	262 (3.4%)	26 (2%)	7 (2.1%)
	**Glasgow coma score**			
	15	5212 (67.4%)	973 (76.3%)	256 (76.9%)
	<15	1484 (19.2%)	211 (16.5%)	45 (13.5%)
	Unknown	1036 (13.4%)	91 (7.1%)	32 (9.6%)
	**Urea (mmol/L)**			
	<7	3316 (42.9%)	603 (47.3%)	181 (54.4%)
	7-14	2110 (27.3%)	346 (27.1%)	82 (24.6%)
	>14	831 (10.7%)	162 (12.7%)	30 (9%)
	Missing	1475 (19.1%)	164 (12.9%)	40 (12%)
	**C-reactive protein (mg/dL)**			
	<50	888 (11.5%)	279 (21.9%)	90 (27%)
	50-99	1269 (16.4%)	245 (19.2%)	67 (20.1%)
	>=100	4438 (57.4%)	609 (47.8%)	132 (39.6%)
	Missing	1137 (14.7%)	142 (11.1%)	44 (13.2%)
**Respiratory support and treatments**	**Respiratory support**			
	Oxygen only	1006 (13%)	260 (20.4%)	78 (23.4%)
	Non-invasive	1768 (22.9%)	461 (36.2%)	157 (47.1%)
	Invasive	4958 (64.1%)	554 (43.5%)	98 (29.4%)
	**Proning**	3110 (40.2%)	402 (31.5%)	61 (18.3%)
	**Extra corporeal memberance oxygenation (ECMO)**	267 (3.5%)	41 (3.2%)	6 (1.8%)
	**Steroids**			
	Yes	2069 (26.8%)	428 (33.6%)	223 (67%)
	No	5210 (67.4%)	794 (62.3%)	104 (31.2%)
	Missing	453 (5.9%)	53 (4.2%)	6 (1.8%)

* Symptom onset summary statistics based on patients with symptoms up to 3 weeks before admission only

Table E4: Baseline characteristics of ward patients presenting in the first wave, divided by 3 equal time points (N=54,632). Disclosure threshold of 5 was used.

Type	Characteristic	1 - Weeks 11 to 17<br>9 Mar to 26 Apr 2020	2 - Weeks 18 to 24<br>27 Apr to 14 Jun 2020	3 - Weeks 25 to 31<br>15 Jun to 2 Aug 2020
	**Total**	39721	12469	2442
Patient characteristics	**Country**			
	England	36353 (91.5%)	11426 (91.6%)	2277 (93.2%)
	Scotland	1680 (4.2%)	481 (3.9%)	33 (1.4%)
	Wales	1688 (4.2%)	562 (4.5%)	132 (5.4%)
	**Age (grouped)**			
	<50	4697 (11.8%)	1397 (11.2%)	407 (16.7%)
	50-69	10090 (25.4%)	2624 (21%)	588 (24.1%)
	70-79	9176 (23.1%)	2771 (22.2%)	521 (21.3%)
	80+	15758 (39.7%)	5677 (45.5%)	926 (37.9%)
	**Age (continuous)**			
	Median (IQR)	76 (IQR=23.5)	78.1 (IQR=22)	74.87 (IQR=25.7)
	Mean (SD)	72 (SD=16.7)	73.7 (SD=17.1)	70 (SD=18.7)
	**Sex**			
	Female	17523 (44.1%)	6194 (49.7%)	1225 (50.2%)
	Male	22198 (55.9%)	6275 (50.3%)	1217 (49.8%)
	**Ethnic group**			
	White	29239 (73.6%)	9963 (79.9%)	1713 (70.1%)
	South Asian	1717 (4.3%)	443 (3.6%)	242 (9.9%)
	East Asian	274 (0.7%)	31 (0.2%)	NA
	Black	1547 (3.9%)	179 (1.4%)	33 (1.4%)
	Other Ethnic Minority	2509 (6.3%)	569 (4.6%)	201 (8.2%)
	Missing	4435 (11.2%)	1284 (10.3%)	253 (10.4%)
	**Number of comorbidities**			
	0	8134 (20.5%)	1755 (14.1%)	450 (18.4%)
	1	9151 (23%)	2145 (17.2%)	466 (19.1%)
	2+	22436 (56.5%)	8569 (68.7%)	1526 (62.5%)
	**AIDS/HIV**	124 (0.3%)	32 (0.3%)	NA
	**Asthma**	5131 (12.9%)	1425 (11.4%)	282 (11.5%)
	**Chronic cardiac disease**	12690 (31.9%)	4495 (36%)	807 (33%)
	**Chronic hematologic disease**	1611 (4.1%)	599 (4.8%)	108 (4.4%)
	**Chronic kidney disease**	6888 (17.3%)	2422 (19.4%)	448 (18.3%)
	**Chronic neurological disorder**	4963 (12.5%)	1742 (14%)	279 (11.4%)
	**Chronic pulmonary disease**	7080 (17.8%)	2310 (18.5%)	421 (17.2%)
	**Dementia**	6851 (17.2%)	2621 (21%)	338 (13.8%)
	**Diabetes Type**			
	Type 1	611 (1.5%)	344 (2.8%)	55 (2.3%)
	Type 2	5212 (13.1%)	2932 (23.5%)	581 (23.8%)
	**Hypertension**	9549 (24%)	5611 (45%)	1047 (42.9%)
	**Malignant neoplasm**	3798 (9.6%)	1394 (11.2%)	295 (12.1%)
	**Malnutrition**	917 (2.3%)	356 (2.9%)	NA
	**Mild to severe liver disease**	1170 (2.9%)	482 (3.9%)	91 (3.7%)
	**Obesity**	3399 (8.6%)	1060 (8.5%)	223 (9.1%)
	**Rheumatologic disorder**	4273 (10.8%)	1572 (12.6%)	287 (11.8%)
	**Health worker**	1737 (4.4%)	561 (4.5%)	62 (2.5%)
Severity of illness	**Asymptomatic**	2262 (5.7%)	2026 (16.2%)	746 (30.5%)
	**Symptom onset (days)***			
	Median (IQR)	3 (IQR=7)	2 (IQR=7)	2 (IQR=7)
	Mean (SD)	4.6 (SD=5.1)	3.9 (SD=5.1)	4 (SD=5)
	**Length of stay (days)**			
	Median (IQR)	7 (IQR=9)	8 (IQR=12)	7 (IQR=11)
	Mean (SD)	10.2 (SD=10.8)	11.6 (SD=11.4)	10.1 (SD=9.6)
	**ISARIC 4C Score**			
	Low (0-3)	1844 (4.6%)	665 (5.3%)	179 (7.3%)
	Intermediate (4-8)	5686 (14.3%)	1697 (13.6%)	417 (17.1%)
	High (9-14)	13895 (35%)	4940 (39.6%)	921 (37.7%)

	Very high (15+)	4604 (11.6%)	1239 (9.9%)	138 (5.7%)
	Missing	13692 (34.5%)	3928 (31.5%)	787 (32.2%)
	**Respiratory rate (breaths/min)**			
	<20	11749 (29.6%)	5018 (40.2%)	1063 (43.5%)
	20-30	20054 (50.5%)	5592 (44.8%)	1027 (42.1%)
	>=30	6055 (15.2%)	1355 (10.9%)	216 (8.8%)
	Missing	1863 (4.7%)	504 (4%)	136 (5.6%)
	**Peripheral oxygen saturation on room air (%)**			
	>=92	29794 (75%)	10129 (81.2%)	2038 (83.5%)
	<92	7953 (20%)	1848 (14.8%)	280 (11.5%)
	Missing	1974 (5%)	492 (3.9%)	124 (5.1%)
	**Glasgow coma score**			
	15	30191 (76%)	9884 (79.3%)	2067 (84.6%)
	<15	5161 (13%)	1700 (13.6%)	205 (8.4%)
	Missing	4369 (11%)	885 (7.1%)	170 (7%)
	**Urea (mmol/L)**			
	<7	14265 (35.9%)	4647 (37.3%)	1014 (41.5%)
	7-14	10675 (26.9%)	3512 (28.2%)	675 (27.6%)
	>14	5741 (14.5%)	1707 (13.7%)	256 (10.5%)
	Missing	9040 (22.8%)	2603 (20.9%)	497 (20.4%)
	**C-reactive protein (mg/dL)**			
	<50	10826 (27.3%)	4496 (36.1%)	947 (38.8%)
	50-99	8131 (20.5%)	2152 (17.3%)	375 (15.4%)
	>=100	13510 (34%)	3127 (25.1%)	552 (22.6%)
	Missing	7254 (18.3%)	2694 (21.6%)	568 (23.3%)
**Respiratory support and treatments**	**Respiratory support**			
	None	9314 (23.4%)	4780 (38.3%)	1184 (48.5%)
	Oxygen only	27017 (68%)	6910 (55.4%)	1143 (46.8%)
	Non-invasive	3390 (8.5%)	779 (6.2%)	115 (4.7%)
	**Steroids**			
	Yes	5285 (13.3%)	1839 (14.7%)	687 (28.1%)
	No	31952 (80.4%)	10081 (80.8%)	1627 (66.6%)
	Missing	2484 (6.3%)	549 (4.4%)	128 (5.2%)
* Symptom onset summary statistics based on patients with symptoms up to 3 weeks before admission only				

Table E5: Baseline characteristics of ICU patients whose maximum respiratory support was oxygen only in the first wave, divided by 3 equal time points (N=1,344). Disclosure threshold of 5 was used.

Type	Characteristic	1 - Weeks 11 to 17<br>9 Mar to 26 Apr 2020	2 - Weeks 18 to 24<br>27 Apr to 14 Jun 2020	3 - Weeks 25 to 31<br>15 Jun to 2 Aug 2020
	**Total**	1006 (100%)	260 (100%)	78 (100%)
Patient characteristics	**Country**			
	England	864 (85.9%)	228 (87.7%)	72 (92.3%)
	Scotland	108 (10.7%)	18 (6.9%)	NA
	Wales	34 (3.4%)	14 (5.4%)	6 (7.7%)
	**Age (grouped)**			
	<50	200 (19.9%)	59 (22.7%)	20 (25.6%)
	50-69	405 (40.3%)	93 (35.8%)	29 (37.2%)
	70-79	217 (21.6%)	40 (15.4%)	29 (37.2%)
	80+	184 (18.3%)	68 (26.2%)	
	**Age (continuous)**			
	Median (IQR)	64.1 (IQR=24)	66.3 (IQR=29.1)	64.6 (IQR=24.1)
	Mean (SD)	64.1 (IQR=16.3)	64.6 (IQR=18.4)	61.4 (IQR=18)
	**Sex**			
	Female	361 (35.9%)	115 (44.2%)	37 (47.4%)
	Male	645 (64.1%)	145 (55.8%)	41 (52.6%)
	**Ethnic group**			
	White	701 (69.7%)	204 (78.5%)	51 (65.4%)
	South Asian	56 (5.6%)	9 (3.5%)	10 (12.8%)
	East Asian	12 (1.2%)	NA	NA
	Black	47 (4.7%)	8 (3.1%)	NA
	Other Ethnic Minority	64 (6.4%)	21 (8.1%)	11 (14.1%)
	Missing	126 (12.5%)	18 (6.9%)	6 (7.7%)
	**Number of comorbidities**			
	0	289 (28.7%)	48 (18.5%)	16 (20.5%)
	1	271 (26.9%)	62 (23.8%)	18 (23.1%)
	2+	446 (44.3%)	150 (57.7%)	44 (56.4%)
	**AIDS/HIV**	9 (0.9%)	NA	NA
	**Asthma**	155 (15.4%)	28 (10.8%)	10 (12.8%)
	**Chronic cardiac disease**	220 (21.9%)	69 (26.5%)	12 (15.4%)
	**Chronic hematologic disease**	38 (3.8%)	11 (4.2%)	NA
	**Chronic kidney disease**	131 (13%)	34 (13.1%)	6 (7.7%)
	**Chronic neurological disorder**	81 (8.1%)	24 (9.2%)	9 (11.5%)
	**Chronic pulmonary disease**	117 (11.6%)	36 (13.8%)	13 (16.7%)
	**Dementia**	70 (7%)	27 (10.4%)	NA
	**Diabetes Type**			
	Type 1	19 (1.9%)	10 (3.8%)	NA
	Type 2	117 (11.6%)	63 (24.2%)	11 (14.1%)
	**Hypertension**	196 (19.5%)	102 (39.2%)	26 (33.3%)
	**Malignant neoplasm**	66 (6.6%)	34 (13.1%)	NA
	**Malnutrition**	17 (1.7%)	9 (3.5%)	NA
	**Mild to severe liver disease**	28 (2.8%)	13 (5%)	NA
	**Obesity**	136 (13.5%)	30 (11.5%)	14 (17.9%)
	**Rheumatologic disorder**	87 (8.6%)	22 (8.5%)	8 (10.3%)
	**Health worker**	70 (7%)	19 (7.3%)	NA
Severity of illness	**Asymptomatic**	25 (2.5%)	29 (11.2%)	18 (23.1%)
	**Symptom onset (days)***			
	Median (IQR)	5 (IQR=8)	3 (IQR=7)	3 (IQR=6)
	Mean (SD)	5.8 (IQR=5.3)	4.6 (IQR=5.7)	3.9 (IQR=4.9)
	**Length of stay (days)**			
	Median (IQR)	9 (IQR=10)	11 (IQR=13)	9 (IQR=10.5)
	Mean (SD)	12.2 (IQR=10.8)	14.2 (IQR=11.8)	11.4 (IQR=7.9)
	**ISARIC 4C Score**			
	Low to intermediate (0-8)	286 (28.4%)	78 (30%)	25 (32.1%)
	High to very high (9+)	371 (36.9%)	99 (38.1%)	26 (33.3%)
	Missing	349 (34.7%)	83 (31.9%)	27 (34.6%)

	**Respiratory rate (breaths/min)**			
	<20	276 (27.4%)	90 (34.6%)	25 (32.1%)
	20-30	514 (51.1%)	130 (50%)	39 (50%)
	>=30	182 (18.1%)	35 (13.5%)	13 (16.7%)
	Missing	34 (3.4%)	5 (1.9%)	1 (1.3%)
	**Peripheral oxygen saturation on room air (%)**			
	>=92	748 (74.4%)	209 (80.4%)	65 (83.3%)
	<92	214 (21.3%)	46 (17.7%)	12 (15.4%)
	Missing	44 (4.4%)	5 (1.9%)	1 (1.3%)
	**Glasgow coma score**			
	15	788 (78.3%)	199 (76.5%)	64 (82.1%)
	<15	97 (9.6%)	38 (14.6%)	8 (10.3%)
	Missing	121 (12%)	23 (8.8%)	6 (7.7%)
	**Urea (mmol/L)**			
	<7	422 (41.9%)	114 (43.8%)	37 (47.4%)
	7-14	242 (24.1%)	62 (23.8%)	17 (21.8%)
	>14	129 (12.8%)	37 (14.2%)	5 (6.4%)
	Missing	213 (21.2%)	47 (18.1%)	19 (24.4%)
	**C-reactive protein (mg/dL)**			
	<50	229 (22.8%)	89 (34.2%)	20 (25.6%)
	50-99	199 (19.8%)	45 (17.3%)	18 (23.1%)
	>=100	431 (42.8%)	86 (33.1%)	23 (29.5%)
	Missing	147 (14.6%)	40 (15.4%)	17 (21.8%)
	**Steroids**			
	Yes	164 (16.3%)	46 (17.7%)	42 (53.8%)
	No	744 (74%)	194 (74.6%)	36 (46.2%)
	Missing	98 (9.7%)	20 (7.7%)	
* Symptom onset summary statistics based on patients with symptoms up to 3 weeks before admission only				

Table E6: Baseline characteristics of ICU patients whose maximum respiratory support was non-invasive ventilation only (including oxygen) in the first wave, divided by 3 equal time points (N=2,386). Disclosure threshold of 5 was used.

Type	Characteristic	1 - Weeks 11 to 17<br>9 Mar to 26 Apr 2020	2 - Weeks 18 to 24<br>27 Apr to 14 Jun 2020	3 - Weeks 25 to 31<br>15 Jun to 2 Aug 2020
	**Total**	1768 (100%)	461 (100%)	157 (100%)
Patient characteristics	**Country**			
	England	1629 (92.1%)	430 (93.3%)	148 (94.3%)
	Scotland	92 (5.2%)	13 (2.8%)	NA
	Wales	47 (2.7%)	18 (3.9%)	9 (5.7%)
	**Age (grouped)**			
	<50	362 (20.5%)	85 (18.4%)	26 (16.6%)
	50-69	837 (47.3%)	218 (47.3%)	67 (42.7%)
	70-79	406 (23%)	116 (25.2%)	64 (40.8%)
	80+	163 (9.2%)	42 (9.1%)	
	**Age (continuous)**			
	Median (IQR)	62 (IQR=20.7)	63.7 (IQR=20.9)	65.9 (IQR=20.3)
	Mean (SD)	61.6 (IQR=14.3)	62.4 (IQR=14.6)	65 (IQR=15.3)
	**Sex**			
	Female	581 (32.9%)	165 (35.8%)	60 (38.2%)
	Male	1187 (67.1%)	296 (64.2%)	97 (61.8%)
	**Ethnic group**			
	White	1211 (68.5%)	348 (75.5%)	113 (72%)
	South Asian	134 (7.6%)	20 (4.3%)	19 (12.1%)
	East Asian	17 (1%)	NA	NA
	Black	76 (4.3%)	17 (3.7%)	NA
	Other Ethnic Minority	161 (9.1%)	29 (6.3%)	18 (11.4%)
	Missing	169 (9.6%)	47 (10.2%)	7 (4.5%)
	**Number of comorbidities**			
	0	522 (29.5%)	80 (17.4%)	19 (12.1%)
	1	482 (27.3%)	120 (26%)	40 (25.5%)
	2+	764 (43.2%)	261 (56.6%)	98 (62.4%)
	**AIDS/HIV**	12 (0.7%)	NA	NA
	**Asthma**	337 (19.1%)	77 (16.7%)	31 (19.7%)
	**Chronic cardiac disease**	321 (18.2%)	91 (19.7%)	45 (28.7%)
	**Chronic hematologic disease**	65 (3.7%)	12 (2.6%)	NA
	**Chronic kidney disease**	162 (9.2%)	50 (10.8%)	20 (12.7%)
	**Chronic neurological disorder**	102 (5.8%)	33 (7.2%)	7 (4.5%)
	**Chronic pulmonary disease**	252 (14.3%)	89 (19.3%)	39 (24.8%)
	**Dementia**	24 (1.4%)	8 (1.7%)	NA
	**Diabetes Type**			
	Type 1	31 (1.8%)	13 (2.8%)	NA
	Type 2	252 (14.3%)	132 (28.6%)	38 (24.2%)
	**Hypertension**	388 (21.9%)	194 (42.1%)	73 (46.5%)
	**Malignant neoplasm**	91 (5.1%)	40 (8.7%)	NA
	**Malnutrition**	20 (1.1%)	5 (1.1%)	NA
	**Mild to severe liver disease**	40 (2.3%)	13 (2.8%)	NA
	**Obesity**	351 (19.9%)	110 (23.9%)	36 (22.9%)
	**Rheumatologic disorder**	150 (8.5%)	41 (8.9%)	6 (3.8%)
	**Health worker**	176 (10%)	74 (16.1%)	NA
Severity of illness	**Asymptomatic**	13 (0.7%)	15 (3.3%)	6 (3.8%)
	**Symptom onset (days)***			
	Median (IQR)	7 (IQR=7)	5 (IQR=6.5)	5 (IQR=6)
	Mean (SD)	7 (IQR=4.9)	5.3 (IQR=4.8)	5.3 (IQR=4.5)
	**Length of stay (days)**			
	Median (IQR)	11 (IQR=9)	11 (IQR=12)	10 (IQR=8)
	Mean (SD)	12.9 (IQR=10)	14.7 (IQR=12.7)	11.7 (IQR=7.9)
	**ISARIC 4C Score**			
	Low to intermediate (0-8)	514 (29.1%)	142 (30.8%)	49 (31.2%)

	High to very high (9+)	735 (41.6%)	229 (49.7%)	80 (51%)
	Missing	519 (29.4%)	90 (19.5%)	28 (17.8%)
	**Respiratory rate (breaths/min)**			
	<20	264 (14.9%)	81 (17.6%)	26 (16.6%)
	20-30	924 (52.3%)	267 (57.9%)	86 (54.8%)
	>=30	529 (29.9%)	110 (23.9%)	44 (28%)
	Missing	51 (2.9%)	3 (0.7%)	1 (0.6%)
	**Peripheral oxygen saturation on room air (%)**			
	>=92	1078 (61%)	294 (63.8%)	101 (64.3%)
	<92	620 (35.1%)	162 (35.1%)	53 (33.8%)
	Missing	70 (4%)	5 (1.1%)	3 (1.9%)
	**Glasgow coma score**			
	15	1498 (84.7%)	405 (87.9%)	132 (84.1%)
	<15	99 (5.6%)	31 (6.7%)	13 (8.3%)
	Missing	171 (9.7%)	25 (5.4%)	12 (7.6%)
	**Urea (mmol/L)**			
	<7	790 (44.7%)	226 (49%)	94 (59.9%)
	7-14	464 (26.2%)	127 (27.5%)	36 (22.9%)
	>14	187 (10.6%)	54 (11.7%)	16 (10.2%)
	Missing	327 (18.5%)	54 (11.7%)	11 (7%)
	**C-reactive protein (mg/dL)**			
	<50	193 (10.9%)	101 (21.9%)	50 (31.8%)
	50-99	361 (20.4%)	93 (20.2%)	24 (15.3%)
	>=100	977 (55.3%)	229 (49.7%)	68 (43.3%)
	Missing	237 (13.4%)	38 (8.2%)	15 (9.6%)
	**Steroids**			
	Yes	363 (20.5%)	160 (34.7%)	112 (71.3%)
	No	1316 (74.4%)	291 (63.1%)	45 (28.7%)
	Missing	89 (5%)	10 (2.2%)	
* Symptom onset summary statistics based on patients with symptoms up to 3 weeks before admission only				

Table E7: Baseline characteristics of ICU patients whose maximum respiratory support was invasive ventilation only (including oxygen) in the first wave, divided by 3 equal time points (N=5,610). Disclosure threshold of 5 was used.

Type	Characteristic	1 - Weeks 11 to 17<br>9 Mar to 26 Apr 2020	2 - Weeks 18 to 24<br>27 Apr to 14 Jun 2020	3 - Weeks 25 to 31<br>15 Jun to 2 Aug 2020
	**Total**	4958 (100%)	554 (100%)	98 (100%)
Patient characteristics	**Country**			
	England	4480 (90.4%)	495 (89.4%)	92 (93.9%)
	Scotland	260 (5.2%)	22 (4%)	NA
	Wales	218 (4.4%)	37 (6.7%)	6 (6.1%)
	**Age (grouped)**			
	<50	1002 (20.2%)	122 (22%)	33 (33.7%)
	50-69	2952 (59.5%)	326 (58.8%)	50 (51%)
	70-79	876 (17.7%)	88 (15.9%)	15 (15.3%)
	80+	128 (2.6%)	18 (3.2%)	
	**Age (continuous)**			
	Median (IQR)	60.4 (IQR=15.8)	59.6 (IQR=16.2)	58.6 (IQR=19.8)
	Mean (SD)	59.5 (IQR=12.2)	58.8 (IQR=13)	56.7 (IQR=14.4)
	**Sex**			
	Female	1372 (27.7%)	185 (33.4%)	31 (31.6%)
	Male	3586 (72.3%)	369 (66.6%)	67 (68.4%)
	**Ethnic group**			
	White	2842 (57.3%)	317 (57.2%)	45 (45.9%)
	South Asian	372 (7.5%)	58 (10.5%)	25 (25.5%)
	East Asian	95 (1.9%)	NA	NA
	Black	345 (7%)	32 (5.8%)	NA
	Other Ethnic Minority	606 (12.2%)	72 (13%)	19 (19.4%)
	Missing	698 (14.1%)	75 (13.5%)	9 (9.2%)
	**Number of comorbidities**			
	0	1844 (37.2%)	131 (23.6%)	22 (22.4%)
	1	1485 (30%)	136 (24.5%)	22 (22.4%)
	2+	1629 (32.9%)	287 (51.8%)	54 (55.1%)
	**AIDS/HIV**	33 (0.7%)	NA	NA
	**Asthma**	750 (15.1%)	82 (14.8%)	13 (13.3%)
	**Chronic cardiac disease**	606 (12.2%)	90 (16.2%)	19 (19.4%)
	**Chronic hematologic disease**	119 (2.4%)	11 (2%)	NA
	**Chronic kidney disease**	279 (5.6%)	45 (8.1%)	9 (9.2%)
	**Chronic neurological disorder**	192 (3.9%)	30 (5.4%)	9 (9.2%)
	**Chronic pulmonary disease**	324 (6.5%)	47 (8.5%)	9 (9.2%)
	**Dementia**	47 (0.9%)	10 (1.8%)	NA
	**Diabetes Type**			
	Type 1	59 (1.2%)	26 (4.7%)	NA
	Type 2	585 (11.8%)	148 (26.7%)	29 (29.6%)
	**Hypertension**	928 (18.7%)	226 (40.8%)	39 (39.8%)
	**Malignant neoplasm**	212 (4.3%)	27 (4.9%)	NA
	**Malnutrition**	34 (0.7%)	5 (0.9%)	NA
	**Mild to severe liver disease**	104 (2.1%)	19 (3.4%)	NA
	**Obesity**	1037 (20.9%)	142 (25.6%)	21 (21.4%)
	**Rheumatologic disorder**	294 (5.9%)	39 (7%)	7 (7.1%)
	**Health worker**	436 (8.8%)	84 (15.2%)	NA
Severity of illness	**Asymptomatic**	40 (0.8%)	30 (5.4%)	13 (13.3%)
	**Symptom onset (days)***			
	Median (IQR)	7 (IQR=6)	6 (IQR=6)	4 (IQR=5)
	Mean (SD)	7.2 (IQR=4.7)	6.1 (IQR=4.9)	5 (IQR=4.4)
	**Length of stay (days)**			
	Median (IQR)	19 (IQR=21)	19 (IQR=18)	18.5 (IQR=15.8)
	Mean (SD)	23.6 (IQR=19.4)	22.3 (IQR=16.6)	19.2 (IQR=12.6)
	**ISARIC 4C Score**			
	Low to intermediate (0-8)	1207 (24.3%)	166 (30%)	33 (33.7%)

	High to very high (9+)	2027 (40.9%)	242 (43.7%)	35 (35.7%)
	Missing	1724 (34.8%)	146 (26.4%)	30 (30.6%)
	**Respiratory rate (breaths/min)**			
	<20	777 (15.7%)	114 (20.6%)	17 (17.3%)
	20-30	2299 (46.4%)	243 (43.9%)	45 (45.9%)
	>=30	1668 (33.6%)	170 (30.7%)	32 (32.7%)
	Missing	214 (4.3%)	27 (4.9%)	4 (4.1%)
	**Peripheral oxygen saturation on room air (%)**			
	>=92	2726 (55%)	338 (61%)	55 (56.1%)
	<92	2020 (40.7%)	195 (35.2%)	39 (39.8%)
	Missing	212 (4.3%)	21 (3.8%)	4 (4.1%)
	**Glasgow coma score**			
	15	2926 (59%)	369 (66.6%)	60 (61.2%)
	<15	1288 (26%)	142 (25.6%)	24 (24.5%)
	Missing	744 (15%)	43 (7.8%)	14 (14.3%)
	**Urea (mmol/L)**			
	<7	2104 (42.4%)	263 (47.5%)	50 (51%)
	7-14	1404 (28.3%)	157 (28.3%)	29 (29.6%)
	>14	515 (10.4%)	71 (12.8%)	9 (9.2%)
	Missing	935 (18.9%)	63 (11.4%)	10 (10.2%)
	**C-reactive protein (mg/dL)**			
	<50	466 (9.4%)	89 (16.1%)	20 (20.4%)
	50-99	709 (14.3%)	107 (19.3%)	25 (25.5%)
	>=100	3030 (61.1%)	294 (53.1%)	41 (41.8%)
	Missing	753 (15.2%)	64 (11.6%)	12 (12.2%)
	**Steroids**			
	Yes	1542 (31.1%)	222 (40.1%)	69 (70.4%)
	No	3150 (63.5%)	309 (55.8%)	29 (29.6%)
	Missing	266 (5.4%)	23 (4.2%)	

* Symptom onset summary statistics based on patients with symptoms up to 3 weeks before admission only

Table E8: Baseline characteristics of ward patients who received no respiratory support in the first wave, divided by 3 equal time points (N=15,278). Disclosure threshold of 5 was used.

Type	Characteristic	1 - Weeks 11 to 17 9 Mar to 26 Apr 2020	2 - Weeks 18 to 24 27 Apr to 14 Jun 2020	3 - Weeks 25 to 31 15 Jun to 2 Aug 2020
	**Total**	9314 (100%)	4780 (100%)	1184 (100%)
Patient characteristics	**Country**			
	England	8324 (89.4%)	4363 (91.3%)	1091 (92.1%)
	Scotland	515 (5.5%)	210 (4.4%)	19 (1.6%)
	Wales	475 (5.1%)	207 (4.3%)	74 (6.2%)
	**Age (grouped)**			
	<50	1700 (18.3%)	791 (16.5%)	242 (20.4%)
	50-69	2415 (25.9%)	974 (20.4%)	287 (24.2%)
	70-79	1880 (20.2%)	986 (20.6%)	214 (18.1%)
	80+	3319 (35.6%)	2029 (42.4%)	441 (37.2%)
	**Age (continuous)**			
	Median (IQR)	73.1 (IQR=28.4)	76.6 (IQR=26.4)	73.6 (IQR=28.9)
	Mean (SD)	68.7 (IQR=19)	71.2 (IQR=19.4)	68 (IQR=20.3)
	**Sex**			
	Female	4474 (48%)	2523 (52.8%)	614 (51.9%)
	Male	4840 (52%)	2257 (47.2%)	570 (48.1%)
	**Ethnic group**			
	White	6530 (70.1%)	3762 (78.7%)	828 (69.9%)
	South Asian	515 (5.5%)	215 (4.5%)	117 (9.9%)
	East Asian	70 (0.8%)	NA	NA
	Black	403 (4.3%)	87 (1.8%)	NA
	Other Ethnic Minority	600 (6.4%)	237 (5%)	112 (9.5%)
	Missing	1196 (12.8%)	479 (10%)	127 (10.7%)
	**Number of comorbidities**			
	0	2667 (28.6%)	1006 (21%)	298 (25.2%)
	1	2018 (21.7%)	848 (17.7%)	225 (19%)
	2+	4629 (49.7%)	2926 (61.2%)	661 (55.8%)
	**AIDS/HIV**	29 (0.3%)	12 (0.3%)	NA
	**Asthma**	1125 (12.1%)	536 (11.2%)	121 (10.2%)
	**Chronic cardiac disease**	2492 (26.8%)	1496 (31.3%)	355 (30%)
	**Chronic hematologic disease**	335 (3.6%)	200 (4.2%)	45 (3.8%)
	**Chronic kidney disease**	1356 (14.6%)	823 (17.2%)	213 (18%)
	**Chronic neurological disorder**	973 (10.4%)	546 (11.4%)	122 (10.3%)
	**Chronic pulmonary disease**	1171 (12.6%)	623 (13%)	134 (11.3%)
	**Dementia**	1436 (15.4%)	907 (19%)	161 (13.6%)
	**Diabetes Type**			
	Type 1	187 (2%)	142 (3%)	NA
	Type 2	1257 (13.5%)	991 (20.7%)	256 (21.6%)
	**Hypertension**	2303 (24.7%)	1996 (41.8%)	461 (38.9%)
	**Malignant neoplasm**	774 (8.3%)	479 (10%)	134 (11.3%)
	**Malnutrition**	215 (2.3%)	130 (2.7%)	NA
	**Mild to severe liver disease**	304 (3.3%)	186 (3.9%)	44 (3.7%)
	**Obesity**	601 (6.5%)	339 (7.1%)	81 (6.8%)
	**Rheumatologic disorder**	900 (9.7%)	545 (11.4%)	123 (10.4%)
	**Health worker**	601 (6.5%)	259 (5.4%)	NA
Severity of illness	**Asymptomatic**	656 (7%)	1069 (22.4%)	446 (37.7%)
	**Symptom onset (days)***			
	Median (IQR)	2 (IQR=7)	1 (IQR=6)	2 (IQR=6)
	Mean (SD)	4 (IQR=5.2)	3.4 (IQR=5.1)	3.7 (IQR=4.9)
	**Length of stay (days)**			
	Median (IQR)	6 (IQR=11)	7 (IQR=13)	6 (IQR=11)
	Mean (SD)	9.9 (IQR=12.5)	10.9 (IQR=12.1)	9.6 (IQR=10.1)
	**ISARIC 4C Score**			
	Low (0-3)	732 (7.9%)	391 (8.2%)	110 (9.3%)
	Intermediate (4-8)	1288 (13.8%)	662 (13.8%)	196 (16.6%)
	High (9-14)	2460 (26.4%)	1626 (34%)	356 (30.1%)

	Very high (15+)	333 (3.6%)	150 (3.1%)	23 (1.9%)
	Missing	4501 (48.3%)	1951 (40.8%)	499 (42.1%)
	**Respiratory rate (breaths/min)**			
	<20	3501 (37.6%)	1624 (34%)	377 (31.8%)
	20-30	604 (6.5%)	180 (3.8%)	50 (4.2%)
	>=30	1268 (13.6%)	423 (8.8%)	117 (9.9%)
	Missing	3501 (37.6%)	1624 (34%)	377 (31.8%)
	**Peripheral oxygen saturation on room air (%)**			
	>=92	7497 (80.5%)	4200 (87.9%)	1044 (88.2%)
	<92	496 (5.3%)	152 (3.2%)	29 (2.4%)
	Missing	1321 (14.2%)	428 (9%)	111 (9.4%)
	**Glasgow coma score**			
	15	6491 (69.7%)	3769 (78.8%)	978 (82.6%)
	<15	758 (8.1%)	467 (9.8%)	66 (5.6%)
	Missing	2065 (22.2%)	544 (11.4%)	140 (11.8%)
	**Urea (mmol/L)**			
	<7	3449 (37%)	1865 (39%)	496 (41.9%)
	7-14	1915 (20.6%)	1082 (22.6%)	257 (21.7%)
	>14	850 (9.1%)	467 (9.8%)	104 (8.8%)
	Missing	3100 (33.3%)	1366 (28.6%)	327 (27.6%)
	**C-reactive protein (mg/dL)**			
	<50	3406 (36.6%)	2053 (42.9%)	509 (43%)
	50-99	1493 (16%)	622 (13%)	130 (11%)
	>=100	1709 (18.3%)	660 (13.8%)	171 (14.4%)
	Missing	2706 (29.1%)	1445 (30.2%)	374 (31.6%)
	**Steroids**			
	Yes	799 (8.6%)	468 (9.8%)	144 (12.2%)
	No	7150 (76.8%)	3934 (82.3%)	940 (79.4%)
	Missing	365 (14.7%)	378 (7.9%)	100 (8.4%)
* Symptom onset summary statistics based on patients with symptoms up to 3 weeks before admission only				

Table E9: Baseline characteristics of ward patients whose maximum respiratory support oxygen only in the first wave, divided by 3 equal time points (N=35,070). Disclosure threshold of 5 was used.

Type	Characteristic	1 - Weeks 11 to 17<br>9 Mar to 26 Apr 2020	2 - Weeks 18 to 24<br>27 Apr to 14 Jun 2020	3 - Weeks 25 to 31<br>15 Jun to 2 Aug 2020
	**Total**	27017 (100%)	6910 (100%)	1143 (100%)
Patient characteristics	**Country**			
	England	24870 (92.1%)	6333 (91.6%)	1081 (94.6%)
	Scotland	1116 (4.1%)	267 (3.9%)	13 (1.1%)
	Wales	1031 (3.8%)	310 (4.5%)	49 (4.3%)
	Age (grouped)			
	<50	2717 (10.1%)	540 (7.8%)	155 (13.6%)
	50-69	6620 (24.5%)	1432 (20.7%)	271 (23.7%)
	70-79	6343 (23.5%)	1566 (22.7%)	265 (23.2%)
	80+	11337 (42%)	3372 (48.8%)	452 (39.5%)
	Age (continuous)			
	Median (IQR)	77 (IQR=22)	79.5 (IQR=19.4)	76.3 (IQR=23.7)
	Mean (SD)	73.1 (IQR=15.9)	75.5 (IQR=15.4)	71.9 (IQR=17.1)
	Sex			
	Female	11762 (43.5%)	3335 (48.3%)	552 (48.3%)
	Male	15255 (56.5%)	3575 (51.7%)	591 (51.7%)
	Ethnic group			
	White	20233 (74.9%)	5604 (81.1%)	802 (70.2%)
	South Asian	1053 (3.9%)	183 (2.6%)	107 (9.4%)
	East Asian	190 (0.7%)	NA	NA
	Black	1007 (3.7%)	81 (1.2%)	NA
	Other Ethnic Minority	1650 (6.1%)	322 (4.6%)	114 (9.9%)
	Missing	2884 (10.7%)	720 (10.4%)	120 (10.5%)
	Number of comorbidities			
	0	4824 (17.9%)	688 (10%)	141 (12.3%)
	1	6305 (23.3%)	1161 (16.8%)	219 (19.2%)
	2+	15888 (58.8%)	5061 (73.2%)	783 (68.5%)
	**AIDS/HIV**	80 (0.3%)	20 (0.3%)	NA
	**Asthma**	3515 (13%)	783 (11.3%)	145 (12.7%)
	**Chronic cardiac disease**	9116 (33.7%)	2698 (39%)	407 (35.6%)
	**Chronic hematologic disease**	1124 (4.2%)	366 (5.3%)	57 (5%)
	**Chronic kidney disease**	5016 (18.6%)	1427 (20.7%)	213 (18.6%)
	**Chronic neurological disorder**	3636 (13.5%)	1096 (15.9%)	145 (12.7%)
	**Chronic pulmonary disease**	5124 (19%)	1467 (21.2%)	251 (22%)
	**Dementia**	5079 (18.8%)	1604 (23.2%)	169 (14.8%)
	**Diabetes Type**			
	Type 1	387 (1.4%)	181 (2.6%)	NA
	Type 2	3477 (12.9%)	1701 (24.6%)	284 (24.8%)
	**Hypertension**	6437 (23.8%)	3217 (46.6%)	530 (46.4%)
	**Malignant neoplasm**	2751 (10.2%)	834 (12.1%)	148 (12.9%)
	**Malnutrition**	639 (2.4%)	209 (3%)	NA
	**Mild to severe liver disease**	763 (2.8%)	262 (3.8%)	42 (3.7%)
	**Obesity**	2362 (8.7%)	592 (8.6%)	126 (11%)
	**Rheumatologic disorder**	2993 (11.1%)	925 (13.4%)	152 (13.3%)
	**Health worker**	1016 (3.8%)	271 (3.9%)	NA
Severity of illness	**Asymptomatic**	588 (2.2%)	588 (8.5%)	202 (17.7%)
	**Symptom onset (days)**			
	Median (IQR)	3 (IQR=7)	2 (IQR=7)	3 (IQR=7)
	Mean (SD)	4.7 (IQR=5.1)	4.1 (IQR=5.1)	4.1 (IQR=4.9)
	**Length of stay (days)**			
	Median (IQR)	7 (IQR=9)	9 (IQR=11)	8 (IQR=11)
	Mean (SD)	10.3 (IQR=10.3)	12.1 (IQR=10.9)	10.7 (IQR=9)
	**ISARIC 4C Score**			
	Low (0-3)	1044 (3.9%)	258 (3.7%)	63 (5.5%)
	Intermediate (4-8)	3891 (14.4%)	914 (13.2%)	210 (18.4%)
	High (9-14)	10095 (37.4%)	2958 (42.8%)	497 (43.5%)

	Very high (15+)	3736 (13.8%)	945 (13.7%)	99 (8.7%)
	Missing	8251 (30.5%)	1835 (26.6%)	274 (24%)
	**Respiratory rate (breaths/min)**			
	<20	7194 (26.6%)	2289 (33.1%)	403 (35.3%)
	20-30	14689 (54.4%)	3543 (51.3%)	587 (51.4%)
	>=30	4612 (17.1%)	1003 (14.5%)	135 (11.8%)
	Missing	522 (1.9%)	75 (1.1%)	18 (1.6%)
	**Peripheral oxygen saturation on room air (%)**			
	>=92	20109 (74.4%)	5413 (78.3%)	924 (80.8%)
	<92	6216 (23%)	1413 (20.4%)	201 (17.6%)
	Missing	692 (2.6%)	84 (1.2%)	18 (1.6%)
	**Glasgow coma score**			
	15	21006 (77.8%)	5482 (79.3%)	991 (86.7%)
	<15	4014 (14.9%)	1114 (16.1%)	126 (11%)
	Missing	1997 (7.4%)	314 (4.5%)	26 (2.3%)
	**Urea (mmol/L)**			
	<7	9644 (35.7%)	2508 (36.3%)	476 (41.6%)
	7-14	7724 (28.6%)	2163 (31.3%)	369 (32.3%)
	>14	4346 (16.1%)	1091 (15.8%)	139 (12.2%)
	Missing	5303 (19.6%)	1148 (16.6%)	159 (13.9%)
	**C-reactive protein (mg/dL)**			
	<50	6785 (25.1%)	2242 (32.4%)	402 (35.2%)
	50-99	5929 (21.9%)	1367 (19.8%)	212 (18.5%)
	>=100	10203 (37.8%)	2141 (31%)	343 (30%)
	Missing	4100 (15.2%)	1160 (16.8%)	186 (16.3%)
**Respiratory support and treatments**	**Steroids**			
	Yes	3831 (14.2%)	1172 (17%)	466 (40.8%)
	No	22232 (82.3%)	5581 (80.8%)	649 (56.8%)
	Missing	954 (3.5%)	157 (2.3%)	28 (2.4%)
* Symptom onset summary statistics based on patients with symptoms up to 3 weeks before admission only				

Table E10: Baseline characteristics of ward patients who received non-invasive ventilation only (including oxygen) in the first wave, divided by 3 equal time points (N=4,284). Disclosure threshold of 5 was used.

Type	Characteristic	1 - Weeks 11 to 17<br>9 Mar to 26 Apr 2020	2 - Weeks 18 to 24<br>27 Apr to 14 Jun 2020	3 - Weeks 25 to 31<br>15 Jun to 2 Aug 2020
	**Total**	3390 (100%)	779 (100%)	115 (100%)
Patient characteristics	**Country**			
	England	3159 (93.2%)	730 (93.7%)	105 (91.3%)
	Scotland	49 (1.4%)	4 (0.5%)	1 (0.9%)
	Wales	182 (5.4%)	45 (5.8%)	9 (7.8%)
	**Age (grouped)**			
	<50	280 (8.3%)	66 (8.5%)	10 (8.7%)
	50-69	1055 (31.1%)	218 (28%)	30 (26.1%)
	70-79	953 (28.1%)	219 (28.1%)	42 (36.5%)
	80+	1102 (32.5%)	276 (35.4%)	33 (28.7%)
	**Age (continuous)**			
	Median (IQR)	74 (IQR=20.3)	75.3 (IQR=19.4)	74.2 (IQR=17.9)
	Mean (SD)	71.5 (IQR=14.3)	72.6 (IQR=14.2)	71.8 (IQR=13.8)
	**Sex**			
	Male	2103 (62%)	443 (56.9%)	56 (48.7%)
	Female	1287 (38%)	336 (43.1%)	59 (51.3%)
	**Ethnic group**			
	White	2476 (73%)	597 (76.6%)	83 (72.2%)
	South Asian	149 (4.4%)	45 (5.8%)	18 (15.7%)
	East Asian	14 (0.4%)	NA	NA
	Black	137 (4%)	11 (1.4%)	NA
	Other Ethnic Minority	259 (7.6%)	41 (5.2%)	8 (6.9%)
	Missing	355 (10.5%)	85 (10.9%)	6 (5.2%)
	**Number of comorbidities**			
	0	643 (19%)	61 (7.8%)	11 (9.6%)
	1	828 (24.4%)	136 (17.5%)	22 (19.1%)
	2+	1919 (56.6%)	582 (74.7%)	82 (71.3%)
	**AIDS/HIV**	15 (0.4%)	NA	NA
	**Asthma**	491 (14.5%)	106 (13.6%)	16 (13.9%)
	**Chronic cardiac disease**	1082 (31.9%)	301 (38.6%)	45 (39.1%)
	**Chronic hematologic disease**	152 (4.5%)	33 (4.2%)	6 (5.2%)
	**Chronic kidney disease**	516 (15.2%)	172 (22.1%)	22 (19.1%)
	**Chronic neurological disorder**	354 (10.4%)	100 (12.8%)	12 (10.4%)
	**Chronic pulmonary disease**	785 (23.2%)	220 (28.2%)	36 (31.3%)
	**Dementia**	336 (9.9%)	110 (14.1%)	8 (7%)
	**Diabetes Type**			
	Type 1	37 (1.1%)	21 (2.7%)	NA
	Type 2	478 (14.1%)	240 (30.8%)	41 (35.7%)
	**Hypertension**	809 (23.9%)	398 (51.1%)	56 (48.7%)
	**Malignant neoplasm**	273 (8.1%)	81 (10.4%)	13 (11.3%)
	**Malnutrition**	63 (1.9%)	17 (2.2%)	NA
	**Mild to severe liver disease**	103 (3%)	34 (4.4%)	5 (4.3%)
	**Obesity**	436 (12.9%)	129 (16.6%)	16 (13.9%)
	**Rheumatologic disorder**	380 (11.2%)	102 (13.1%)	12 (10.4%)
	**Health worker**	120 (3.5%)	31 (4%)	NA
Severity of illness	**Asymptomatic**	49 (1.4%)	35 (4.5%)	7 (6.1%)
	**Symptom onset (days)***			
	Median (IQR)	4 (IQR=7)	3 (IQR=7)	3 (IQR=7)
	Mean (SD)	5.3 (IQR=5.2)	4.7 (IQR=5.4)	5.1 (IQR=5.8)
	**Length of stay (days)**			
	Median (IQR)	8 (IQR=9)	8 (IQR=10)	8 (IQR=10)
	Mean (SD)	10.5 (IQR=9)	11.9 (IQR=10.9)	10.8 (IQR=10)
	**ISARIC 4C Score**			
	Low (0-3)	65 (1.9%)	15 (1.9%)	6 (5.2%)

	Intermediate (4-8)	498 (14.7%)	120 (15.4%)	10 (8.7%)
	High (9-14)	1305 (38.5%)	350 (44.9%)	67 (58.3%)
	Very high (15+)	502 (14.8%)	138 (17.7%)	15 (13%)
	Missing	1020 (30.1%)	156 (20%)	17 (14.8%)
	**Respiratory rate (breaths/min)**			
	<20	614 (18.1%)	176 (22.6%)	20 (17.4%)
	20-30	1864 (55%)	425 (54.6%)	63 (54.8%)
	>=30	838 (24.7%)	172 (22.1%)	31 (27%)
	Missing	74 (2.2%)	6 (0.8%)	1 (0.9%)
	**Peripheral oxygen saturation on room air (%)**			
	>=92	2187 (64.5%)	516 (66.2%)	70 (60.9%)
	<92	1101 (32.5%)	256 (32.9%)	44 (38.3%)
	Missing	102 (3%)	7 (0.9%)	1 (0.9%)
	**Glasgow coma score**			
	15	2694 (79.5%)	633 (81.3%)	98 (85.2%)
	<15	389 (11.5%)	119 (15.3%)	13 (11.3%)
	Missing	307 (9.1%)	27 (3.5%)	4 (3.5%)
	**Urea (mmol/L)**			
	<7	1172 (34.6%)	274 (35.2%)	42 (36.5%)
	7-14	1036 (30.6%)	267 (34.3%)	49 (42.6%)
	>14	545 (16.1%)	149 (19.1%)	13 (11.3%)
	Missing	637 (18.8%)	89 (11.4%)	11 (9.6%)
	**C-reactive protein (mg/dL)**			
	<50	635 (18.7%)	201 (25.8%)	36 (31.3%)
	50-99	709 (20.9%)	163 (20.9%)	33 (28.7%)
	>=100	1598 (47.1%)	326 (41.8%)	38 (33%)
	Missing	448 (13.2%)	89 (11.4%)	8 (7%)
**Respiratory support and treatments**	**Steroids**			
	Yes	655 (19.3%)	199 (25.5%)	77 (67%)
	No	2570 (75.8%)	566 (72.7%)	38 (33%)
	Missing	165 (4.9%)	14 (1.8%)	NA
* Symptom onset summary statistics based on patients with symptoms up to 3 weeks before admission only				

Table E11: In-patient mortality and 95% CIs for 3 equal time-periods (Weeks 11 to 17, 18 to 24 and 25 to 31). 95% confidence intervals calculated by the Exact method. Denominators found in Table 1 and Tables E3-10.

			**1 - Weeks 11 to 17** 9 Mar to 26 Apr 2020		**2 - Weeks 18 to 24** 27 Apr to 14 Jun 2020		**3 - Weeks 25 to 31** 15 Jun to 2 Aug 2020	
**Threshold of care**	**Respiratory support**	**Age group**	**Mortality N (%)**	**95% CI**	**Mortality N (%)**	**95% CI**	**Mortality N (%)**	**95% CI**
**Overall**		**Overall**	15310 (32.3)	(31.8, 32.7)	3416 (24.9)	(24.1, 25.6)	455 (16.4)	(15, 17.8)
		<50	382 (6.1)	(5.5, 6.7)	60 (3.6)	(2.8, 4.6)	15 (3.1)	(1.7, 5)
		50-69	2935 (20.5)	(19.9, 21.2)	488 (15)	(13.8, 16.2)	78 (10.6)	(8.5, 13.1)
		70-79	4126 (38.7)	(37.7, 39.6)	844 (28)	(26.4, 29.6)	123 (20.8)	(17.6, 24.4)
		80+	7867 (48.5)	(47.7, 49.2)	2024 (34.9)	(33.6, 36.1)	239 (24.8)	(22.1, 27.6)
**ICU**	**Invasive**	**Overall**	2034 (41)	(39.7, 42.4)	209 (37.7)	(33.7, 41.9)	41 (41.8)	(31.9, 52.2)
		<50	206 (20.6)	(18.1, 23.2)	25 (20.5)	(13.7, 28.7)	7 (21.2)	(9, 38.9)
		50-69	1182 (40)	(38.3, 41.8)	116 (35.6)	(30.4, 41)	24 (48)	(33.7, 62.6)
		70-79	552 (63)	(59.7, 66.2)	56 (63.6)	(52.7, 73.6)	9 (69.2)	(38.6, 90.9)
		80+	94 (73.4)	(64.9, 80.9)	12 (66.7)	(41, 86.7)	<5	
	**Non-invasive**	**Overall**	587 (33.2)	(31, 35.5)	118 (25.6)	(21.7, 29.8)	39 (24.8)	(18.3, 32.4)
		<50	28 (7.7)	(5.2, 11)	<5		<5	
		50-69	198 (23.7)	(20.8, 26.7)	35 (16.1)	(11.4, 21.6)	13 (19.4)	(10.8, 30.9)
		70-79	229 (56.4)	(51.4, 61.3)	50 (43.1)	(33.9, 52.6)	16 (43.2)	(27.1, 60.5)
		80+	132 (81)	(74.1, 86.7)	29 (69)	(52.9, 82.4)	7 (25.9)	(11.1, 46.3)
	**Oxygen only**	**Overall**	228 (22.7)	(20.1, 25.4)	51 (19.6)	(15, 25)	10 (12.8)	(6.3, 22.3)
		<50	5 (2.5)	(0.8, 5.7)	<5	(1.1, 14.1)	0 (0)	(0, 16.8)
		50-69	67 (16.5)	(13.1, 20.5)	13 (14)	(7.7, 22.7)	<5	
		70-79	77 (35.5)	(29.1, 42.2)	10 (25)	(12.7, 41.2)	<5	
		80+	79 (42.9)	(35.7, 50.4)	25 (36.8)	(25.4, 49.3)	<5	
**Ward**	**None**	**Overall**	1339 (14.4)	(13.7, 15.1)	403 (8.4)	(7.7, 9.3)	71 (6)	(4.7, 7.5)
		<50	20 (1.2)	(0.7, 1.8)	6 (0.8)	(0.3, 1.6)	<5	
		50-69	154 (6.4)	(5.4, 7.4)	39 (4)	(2.9, 5.4)	5 (1.7)	(0.6, 4)
		70-79	313 (16.6)	(15, 18.4)	92 (9.3)	(7.6, 11.3)	12 (5.6)	(2.9, 9.6)
		80+	852 (25.7)	(24.2, 27.2)	266 (13.1)	(11.7, 14.7)	53 (12)	(9.1, 15.4)
	**Non-invasive**	**Overall**	1626 (48)	(46.3, 49.7)	389 (49.9)	(46.4, 53.5)	51 (44.3)	(35.1, 53.9)
		<50	24 (8.6)	(5.6, 12.5)	5 (7.6)	(2.5, 16.8)	0 (0)	(0, 30.8)
		50-69	331 (31.4)	(28.6, 34.3)	67 (30.7)	(24.7, 37.3)	8 (26.7)	(12.3, 45.9)
		70-79	538 (56.5)	(53.2, 59.6)	130 (59.4)	(52.5, 65.9)	18 (42.9)	(27.7, 59)
		80+	733 (66.5)	(63.6, 69.3)	187 (67.8)	(61.9, 73.2)	25 (75.8)	(57.7, 88.9)
	**Oxygen only**	**Overall**	9496 (35.1)	(34.6, 35.7)	2246 (32.5)	(31.4, 33.6)	243 (21.3)	(18.9, 23.7)
		<50	99 (3.6)	(3, 4.4)	17 (3.1)	(1.8, 5)	<5	
		50-69	1003 (15.2)	(14.3, 16)	218 (15.2)	(13.4, 17.2)	25 (9.2)	(6.1, 13.3)
		70-79	2417 (38.1)	(36.9, 39.3)	506 (32.3)	(30, 34.7)	64 (24.2)	(19.1, 29.8)
		80+	5977 (52.7)	(51.8, 53.6)	1505 (44.6)	(42.9, 46.3)	150 (33.2)	(28.9, 37.7)

Appendix 3: Mediation model

Table E12

A: Natural effects mediation model for respiratory support/ICU ward. Exposure: week admission (continuous). Confounders: age, sex, deprivation, comorbidity, severity (respiratory rate, oxygen saturations, GCS, serum urea, serum CRP). Mediators: respiratory support*icu/ward. Exposure-mediator interaction included. 10 imputed datasets used, and results combined using Rubin's rules.

	estimate	standard error	Z	P	exp (estimate)	L95	U95
Pure natural direct effect	-0.167	0.014	-12.280	<0.001	0.847	0.824	0.869
Total natural direct effect	-0.183	0.013	-14.487	<0.001	0.833	0.812	0.853
Pure natural indirect effect	-0.043	0.005	-9.581	<0.001	0.958	0.949	0.966
Total natural indirect effect	-0.060	0.003	-19.587	<0.001	0.942	0.936	0.947
Total effect	-0.226	0.014	-16.215	<0.001	0.797	0.776	0.819

Approximate proportion mediated on risk difference scale = 24.3%.

B: Natural effects mediation model steroid use. Exposure: week admission (continuous). Confounders: age, sex, deprivation, comorbidity, severity (respiratory rate, oxygen saturations, GCS, serum urea, serum CRP). Mediators: respiratory support*icu/ward. Exposure-mediator interaction included. 10 imputed datasets used, and results combined using Rubin's rules.

	estimate	standard error	Z	P	exp (estimate)	L95	U95
Pure natural direct effect	-0.289	0.013	-22.583	<0.001	0.749	0.731	0.768
Total natural direct effect	-0.285	0.013	-22.756	<0.001	0.752	0.733	0.77
Pure natural indirect effect	0.005	0.002	2.641	0.008	1.005	1.001	1.008
Total natural indirect effect	0.008	0.001	6.606	<0.001	1.008	1.006	1.011
Total effect	-0.281	0.013	-21.842	<0.001	0.755	0.736	0.774

Approximate proportion mediated on risk difference scale = -2.5%

C: Natural effects mediation joint model for respiratory support/ICU ward and steroid use. Exposure: week admission (continuous). Confounders: age, sex, deprivation, comorbidity, severity (respiratory rate, oxygen saturations, GCS, serum urea, serum CRP). Mediators: respiratory support*icu/ward*steroid use. Exposure-mediator interaction included. 10 imputed datasets used, and results combined using Rubin's rules.

	estimate	standard error	Z	P	exp (estimate)	L95	U95
Pure natural direct effect	-0.722	0.026	-12.357	<0.001	0.486	0.685	0.760
Total natural direct effect	-0.684	0.022	-17.087	<0.001	0.504	0.655	0.715
Pure natural indirect effect	-0.934	0.010	-6.695	<0.001	0.393	0.916	0.953
Total natural indirect effect	-0.886	0.007	-17.187	<0.001	0.412	0.874	0.898
Total effect	-0.639	0.025	-17.808	<0.001	0.528	0.609	0.672

Approximate proportion mediated on risk difference scale = 22.8%.

Figures

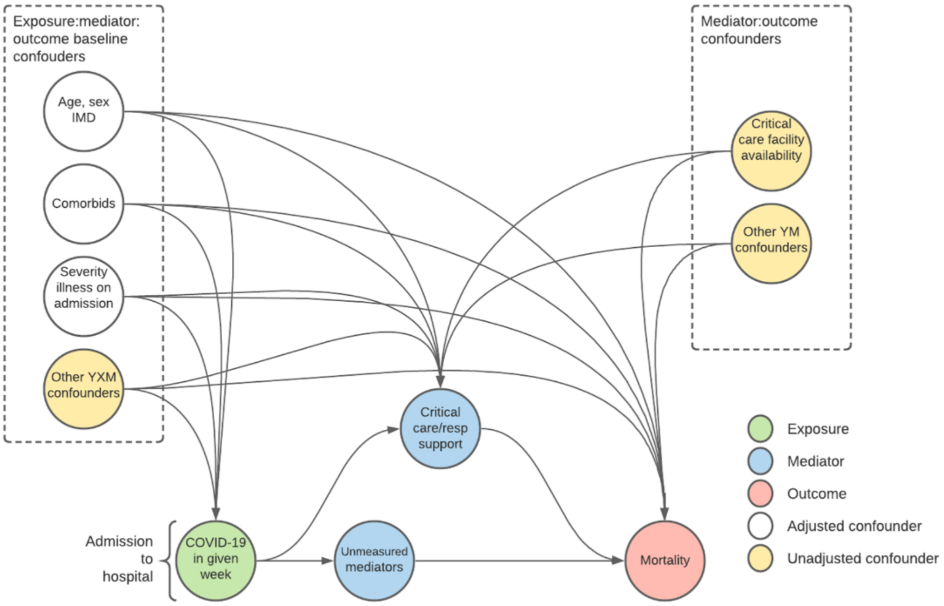

Figure E1: Directed acyclic graph showing putative causal model for analysis.

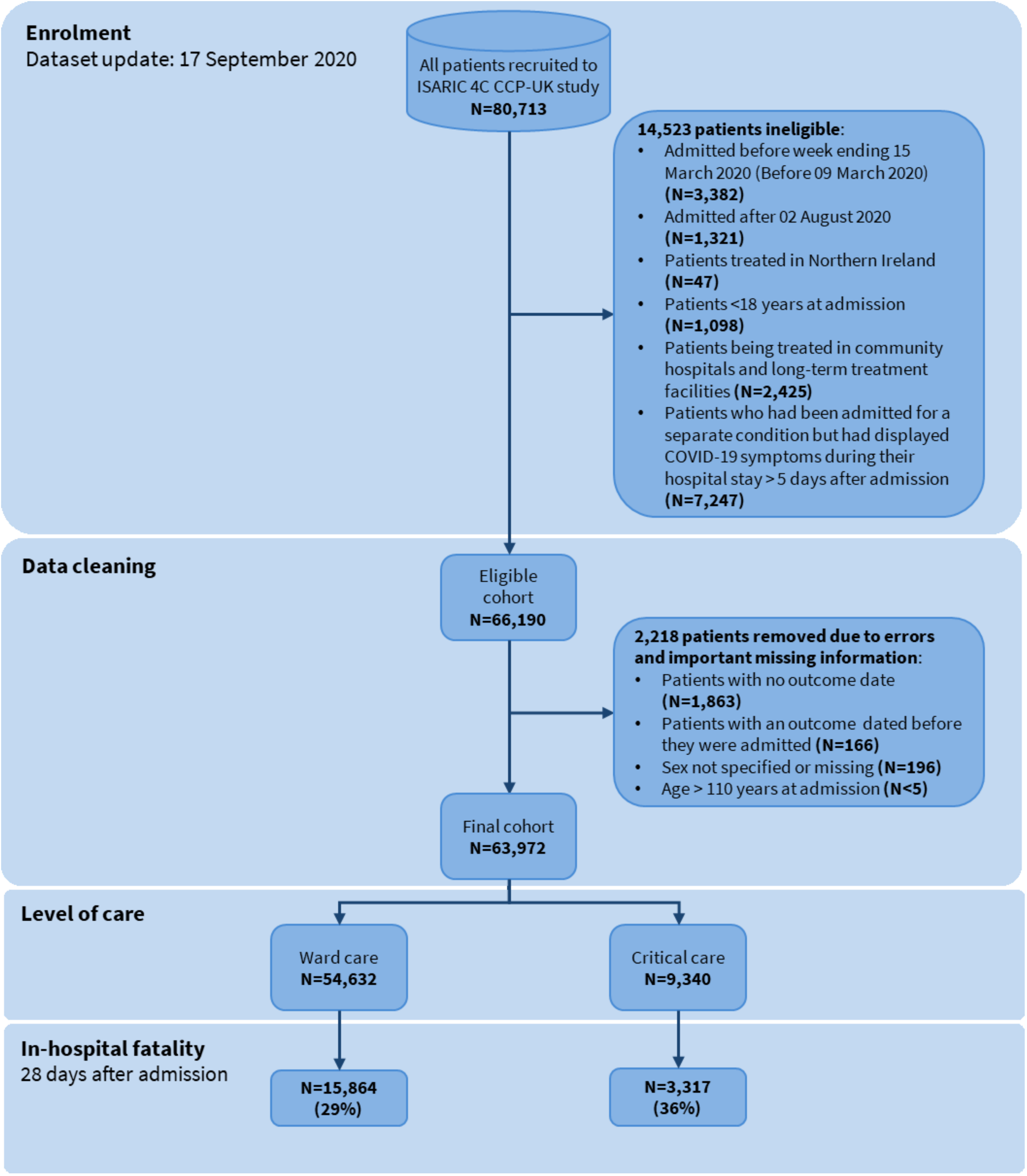

Figure E2: Consort diagram

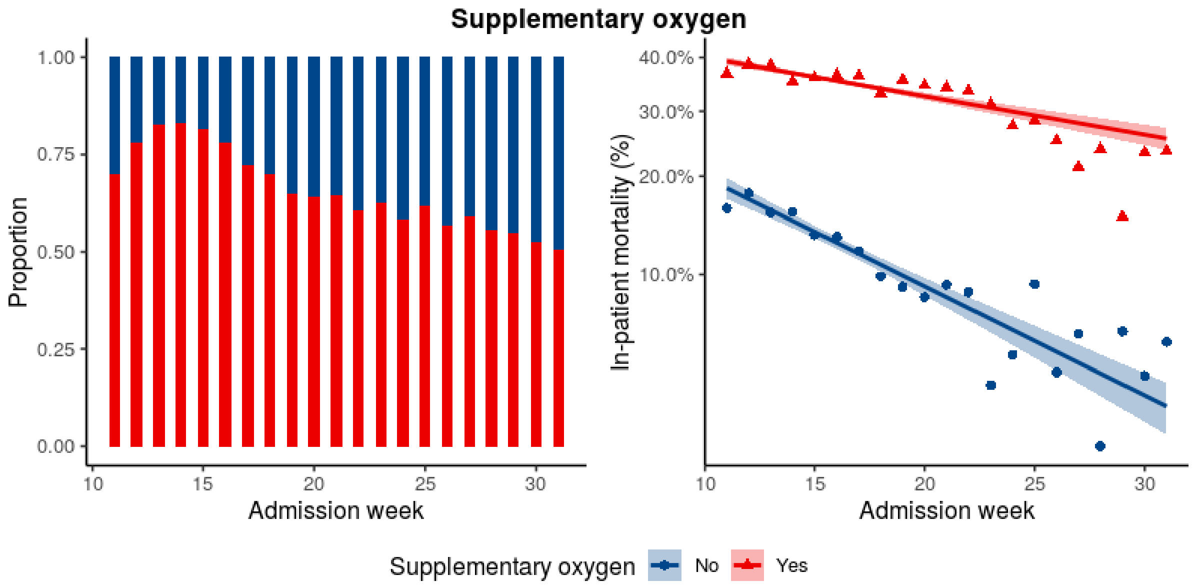

Figure E3: (Left) Changes in the proportions of all patients who received supplementary oxygen over time. (Right) In-hospital mortality rate per category over time

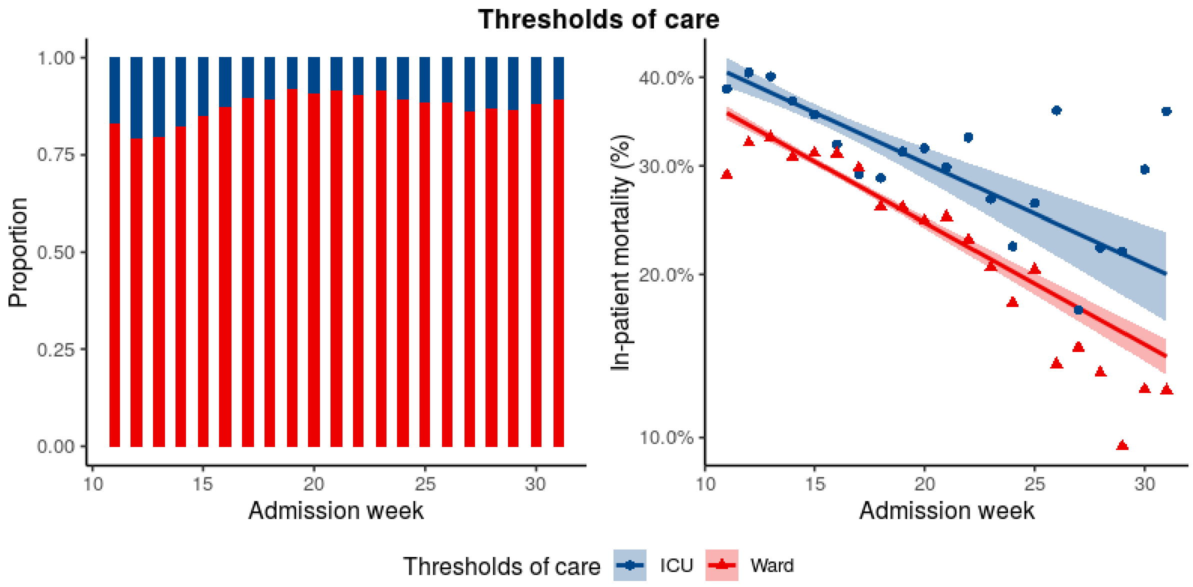

Figure E4: (Left) Changes in the proportions of threshold of care (ICU/Ward) over time. (Right) In-hospital mortality rate per category over time

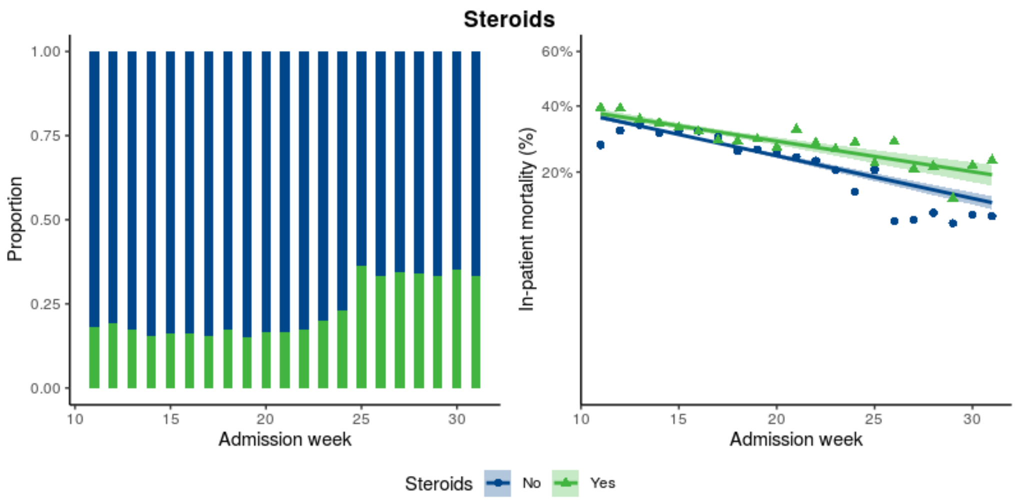

Figure E5: (Left) Changes in the proportions of all patients who received steroids over time. (Right) In-hospital mortality rate per category over time. Unknown measurements are excluded from this figure.

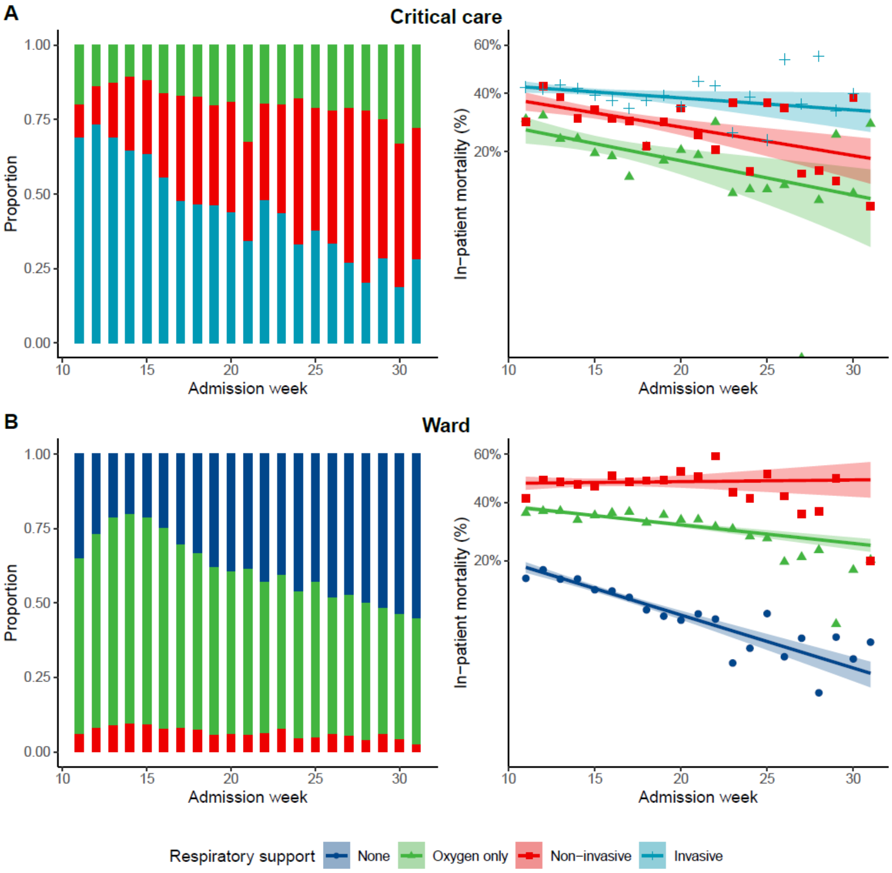

Figure E6: Respiratory support within Critical Care (top) and ward (bottom). (Left) Proportion of respiratory support treatments by week of admission. (Right) Unadjusted in-hospital mortality rate per category by week of admission.

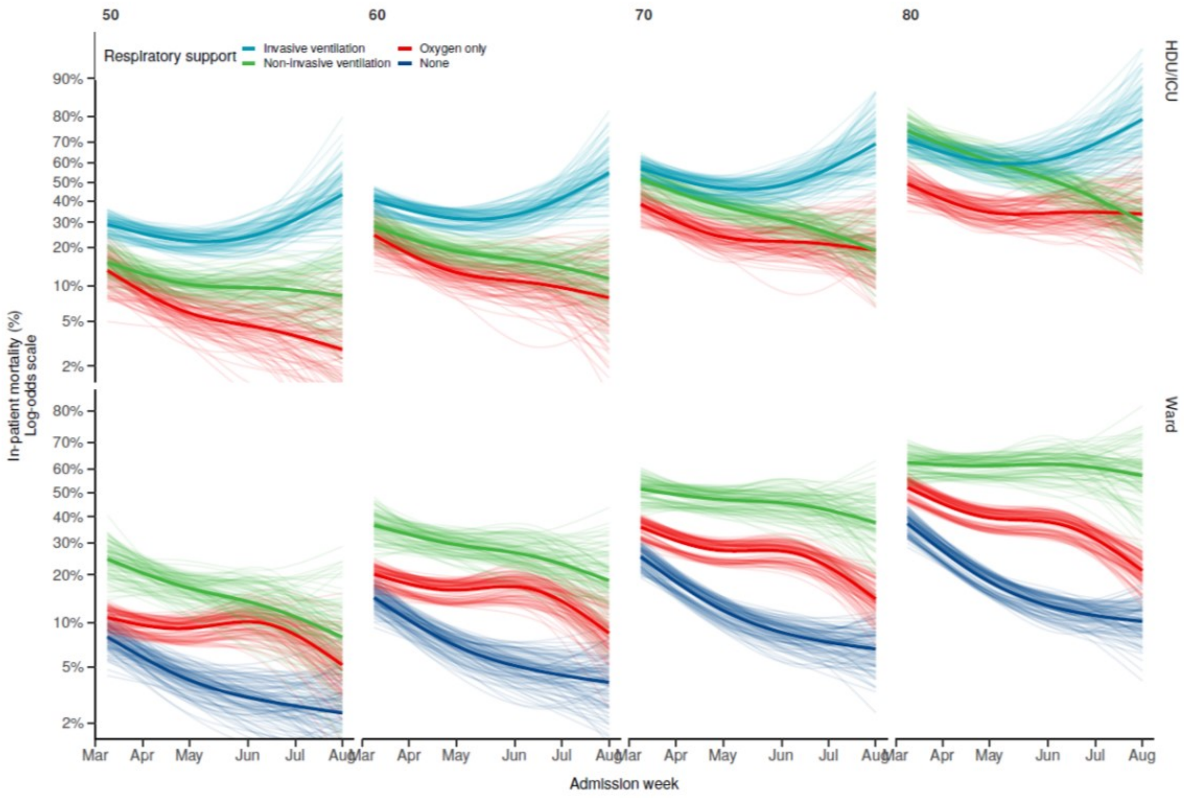

Figure E7: Mortality in adults admitted to hospital with COVID-19 stratified by respiratory support and age. Bayesian generalised additive model, adjusted for age, sex, comorbidity, GCS, respiratory rate, SpO_2, serum urea and CRP, with 3-way interaction between age, week of admission, and level of respiratory support. Missing data imputed with 10 datasets. Plot lines represent samples from posterior distribution.

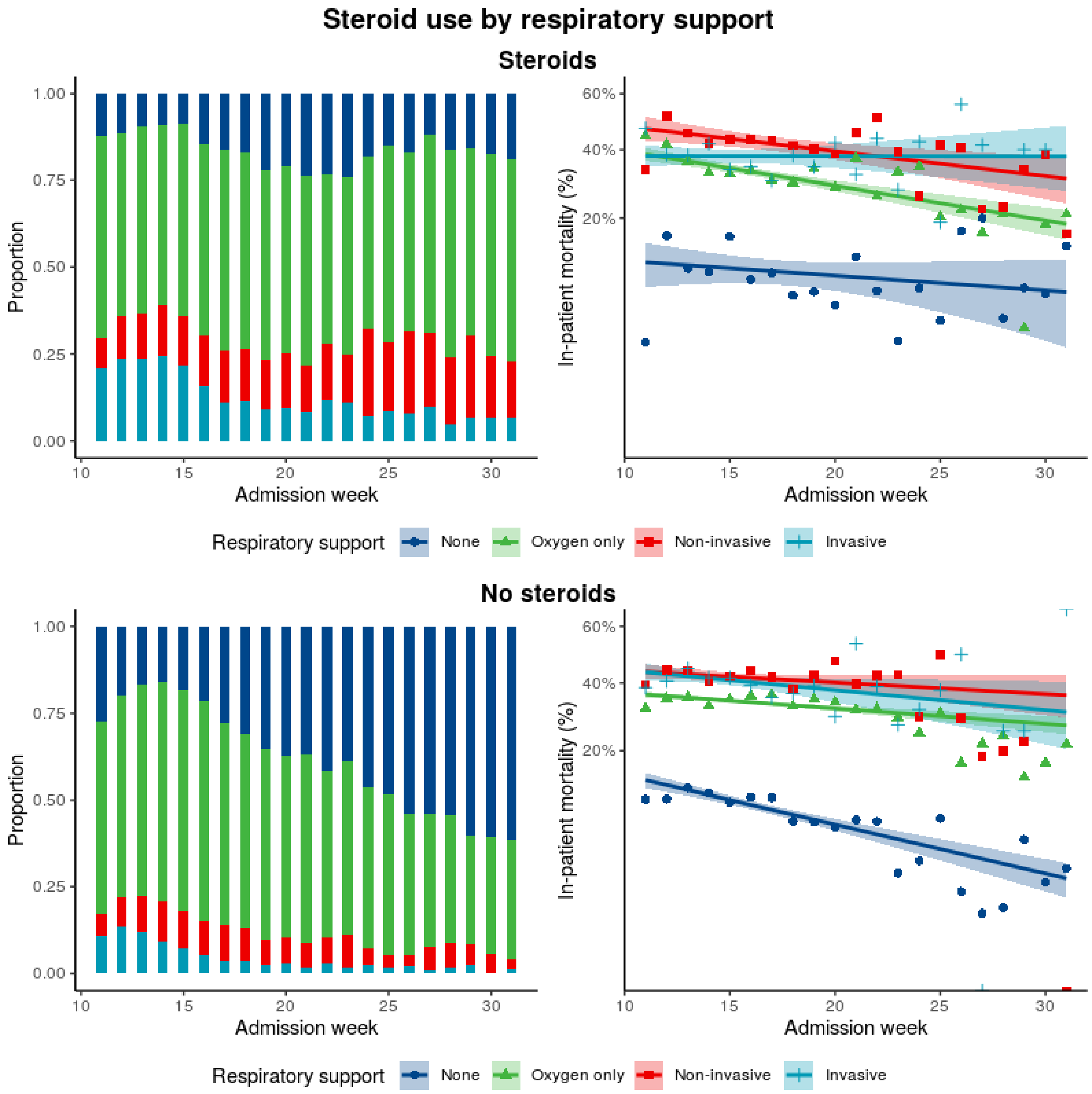

Figure E6: Respiratory support within steroid users (top) and non-steroid users (bottom). (Left) Proportion of respiratory support treatments by week of admission. (Right) In-hospital mortality rate per category by week of admission.

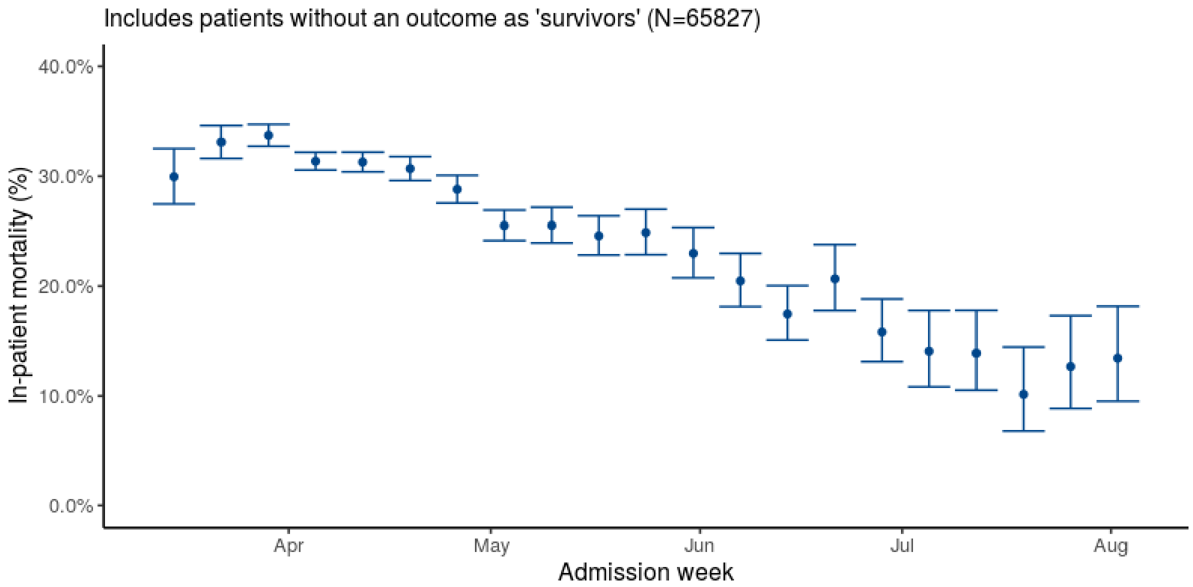

Figure E7: Sensitivity analysis - unadjusted weekly in-hospital mortality and 95% confidence intervals for patients admitted with SARS-CoV-2 from 9 March 2020 to 2 August 2020 Includes those without an outcome dated as 'survivors'. Divided into 3 equal time periods (Weeks 11 to 17, 18 to 24 and 25 to 31). Confidence intervals calculated via exact method.

Appendix 4: Hospital mortality including patients who died in hospital after 28 days

All deaths (including mortality after 28 days)

- Total number of deaths = 20174 (31.5%)
- Total number of deaths within 28 days of admission = 19181 (30%)
- Therefore, 95% of all deaths in the cohort were captured when using a cut off of 28 days after admission.

Plots:

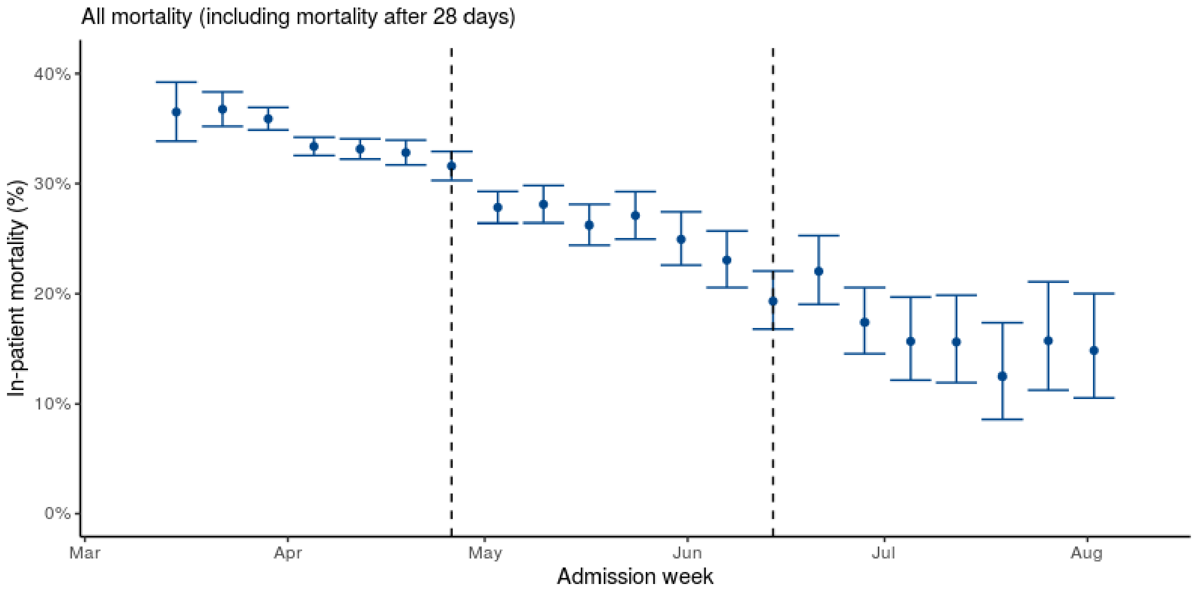

Total mortality over time

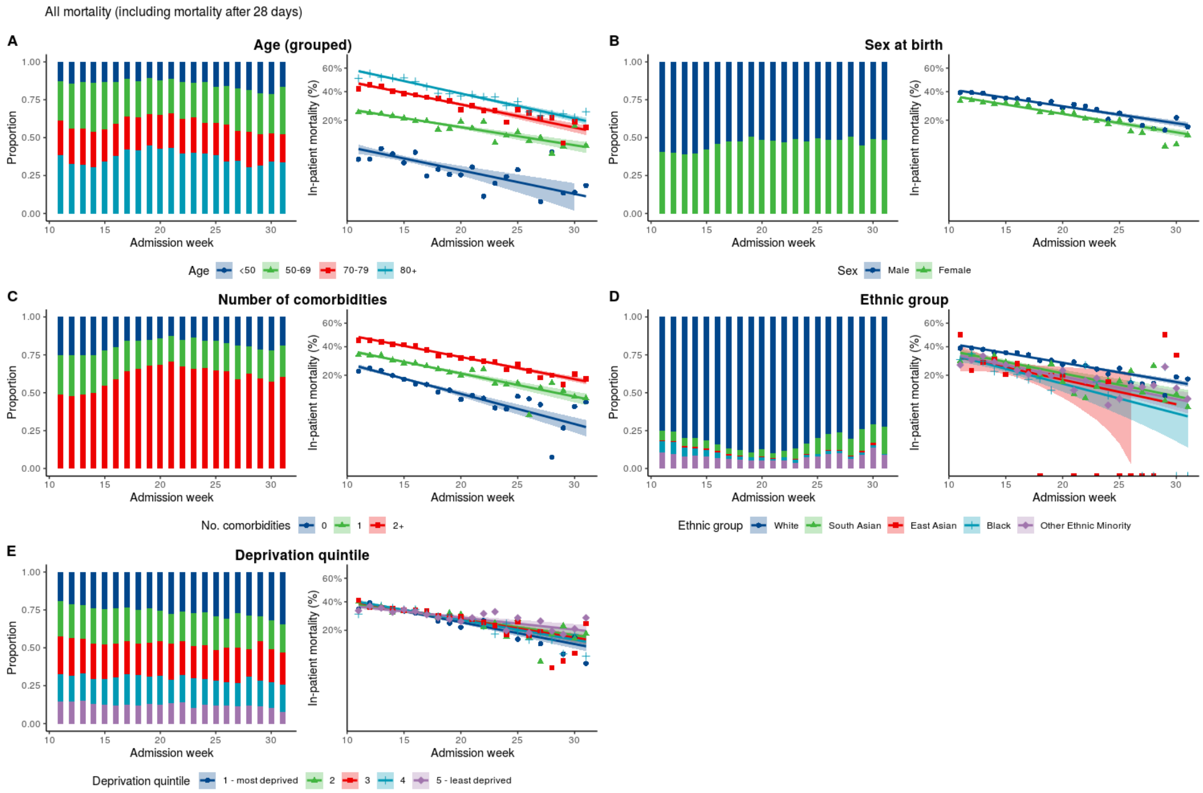

All mortality by demographics

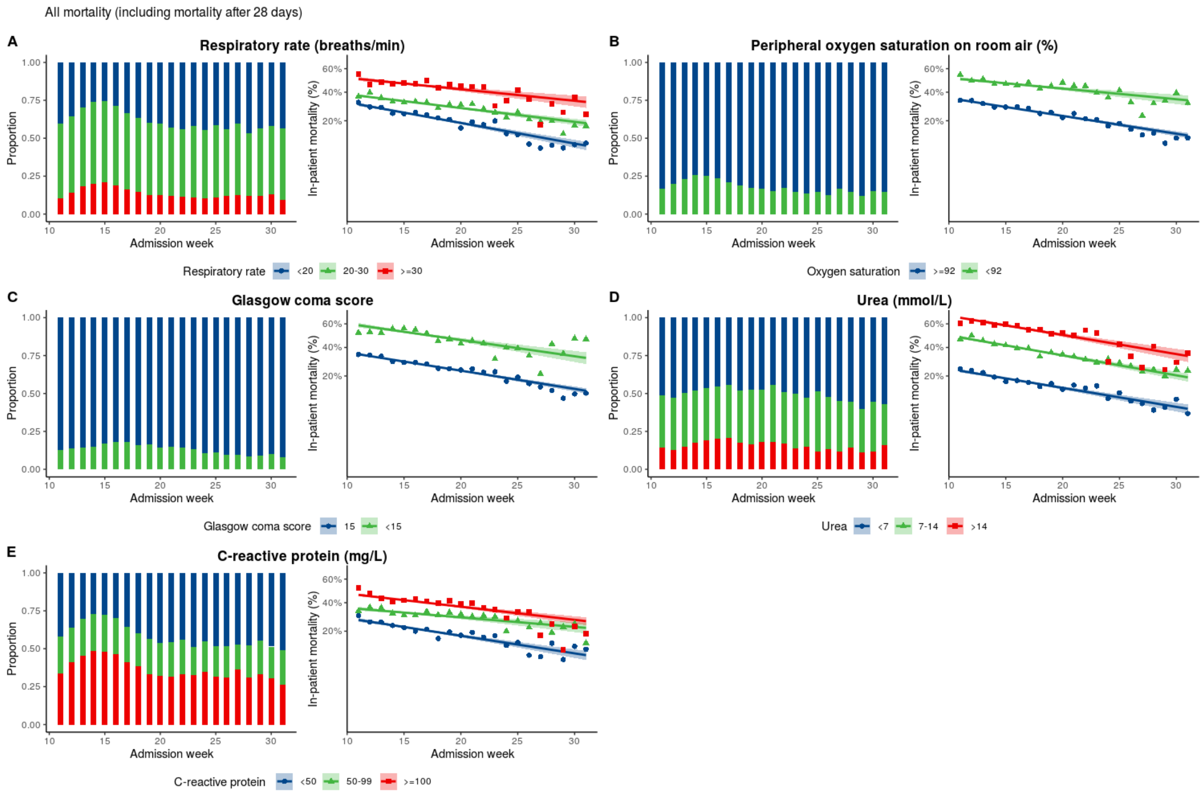

All mortality by severity of illness

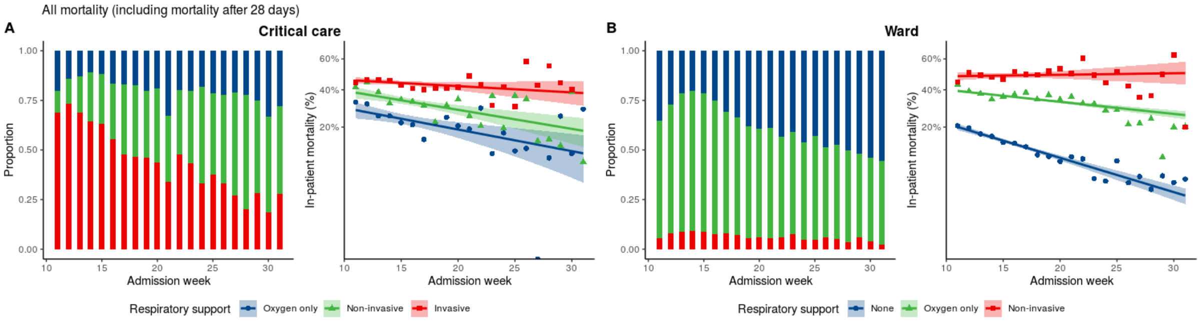

All mortality by respiratory support split by critical care and ward

Appendix 5: Missing data

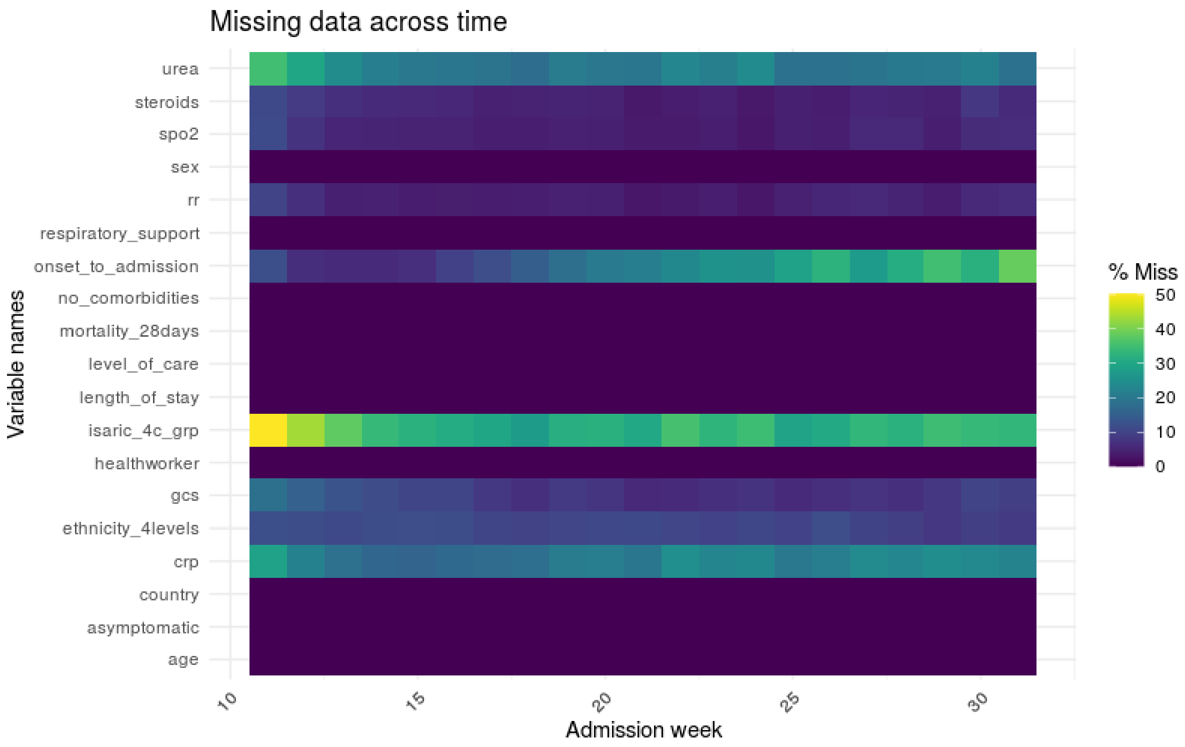

Variable names: age = Age; asymptomatic = Asymptomatic indicator; country = Country; crp = C-reactive protein; ethnicity_4levels = Ethnicity; gca = Glasgow coma score; healthwork_erterm = Healthworker indicator; isaric_4c_grp = ISARIC-4C Score group; length_of_stay = Length of stay; level_of_care = Level of care (Critical care/ICU); mortality_28days = Mortality within 28 days; no_comorbidities = Number of comorbidities; onset_to_admission = Onset to admission; respiratory_support = Respiratory support; rr = Respiratory rate; sex = Sex; spo2 = Peripheral oxygen saturation on room air; steroids = Steroids indicator; urea = Urea.

Correlation between missingess in severity of illness

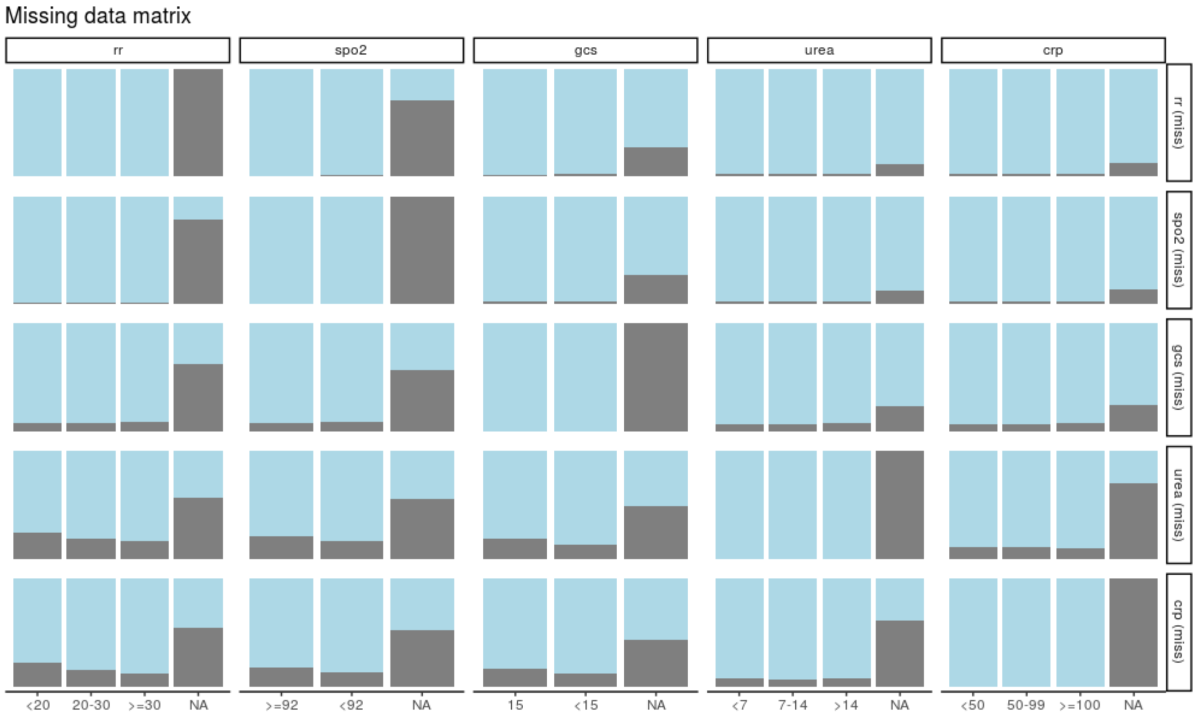

Variable names: rr = Respiratory rate; spo2 = Peripheral oxygen saturation on room air; gca = Glasgow coma score; urea = Urea; crp = C-reactive protein.

ISARIC 4C Score

Type	Characteristic	ISARIC 4C Score N (%) or Mean (SD)	
		Not missing	Missing
	**Total**		
**Outcome**	**Mortality within 28 days**	12996 (30.6)	6185 (28.8)
**Patient characteristics**	**Country**		
	England	38716 (91.2)	19778 (92.0)
	Scotland	1854 (4.4)	853 (4.0)
	Wales	1902 (4.5)	869 (4.0)
	**Age (grouped)**		
	<50	5340 (12.6)	3070 (14.3)
	50-69	12406 (29.2)	5873 (27.3)
	70-79	9508 (22.4)	4772 (22.2)
	80+	15218 (35.8)	7785 (36.2)
	**Age (continuous)**		
	Mean (SD)	70.8 (16.6)	70.3 (17.6)
	**Sex**		
	Female	18304 (43.1)	9545 (44.4)
	Male	24168 (56.9)	11955 (55.6)
	**Ethnic group**		
	White	31208 (73.5)	15539 (72.3)
	South Asian	2043 (4.8)	1062 (4.9)
	East Asian	282 (0.7)	169 (0.8)
	Black	1432 (3.4)	860 (4.0)
	Other Ethnic Minority	2829 (6.7)	1421 (6.6)
	Missing	4678 (11.0)	2449 (11.4)
	**Number of comorbidities**		
	0	7635 (18.0)	5675 (26.4)
	1	9489 (22.3)	4909 (22.8)
	2+	25348 (59.7)	10916 (50.8)
	**Health worker**	2275 (5.4)	959 (4.5)

Type	Characteristic	ISARIC 4C Score N (%) or Mean (SD)	
		Not missing	Missing
Severity of illness	**Asymptomatic**	1954 (4.6)	1875 (8.7)
	**Symptom onset (days)***		
	Mean (SD)	5.0 (5.1)	4.3 (5.2)
	**Length of stay (days)**		
	Mean (SD)	11.4 (11.8)	12.6 (13.4)
	**Respiratory rate (breaths/min)**		
	<20	12516 (29.5)	6984 (32.5)
	20-30	22324 (52.6)	8896 (41.4)
	>=30	7632 (18.0)	2776 (12.9)
	Missing		2844 (13.2)
	**Peripheral oxygen saturation on room air (%)**		
	>=92	32739 (77.1)	14835 (69.0)
	<92	9733 (22.9)	3536 (16.4)
	Missing	0 (0.0)	3129 (14.6)
	**Glasgow coma score**		
	15	35580 (83.8)	13003 (60.5)
	<15	6892 (16.2)	1914 (8.9)
	Missing	0 (0.0)	6583 (30.6)
	**Urea (mmol/L)**		
	<7	20458 (48.2)	3568 (16.6)
	7-14	14730 (34.7)	2670 (12.4)
	>14	7284 (17.2)	1443 (6.7)
	Missing	0 (0.0)	13819 (64.3)
	**C-reactive protein (mg/dL)**		
	<50	14308 (33.7)	3218 (15.0)
	50-99	10026 (23.6)	2213 (10.3)
	>=100	18138 (42.7)	4230 (19.7)
	Missing	0 (0.0)	11839 (55.1)
Respiratory support and treatments	**Level of care**		
	Critical care	6344 (14.9)	2996 (13.9)
	Ward	36128 (85.1)	18504 (86.1)
	**Respiratory support**		
	None	8327 (19.6)	6951 (32.3)
	Oxygen only	25595 (60.3)	10819 (50.3)
	Non-invasive	4840 (11.4)	1830 (8.5)
	Invasive	3710 (8.7)	1900 (8.8)
	**Steroids**		
	Yes	7534 (17.7)	2997 (13.9)
	No	33621 (79.2)	16147 (75.1)
	Missing	1317 (3.1)	2356 (11.0)

References

1. Rickman H.M, Rampling T, Shaw K et al. Nosocomial Transmission of Coronavirus Disease 2019: A Retrospective Study of 66 Hospital-acquired Cases in a London Teaching Hospital. Clinical Infectious Diseases. 2020. ciaa816. Available from: doi:1093/cid/ciaa816.
2. Barrio, I., Arostegui, I., Quintana, J.M. et al. Use of generalised additive models to categorise continuous variables in clinical prediction. BMC Med Res Methodol 13, 83 (2013). https://doi.org/10.1186/1471-2288-13-83
3. Knight SR, Ho A, Pius R, et al. Risk stratification of patients admitted to hospital with covid-19 using the ISARIC WHO Clinical Characterisation Protocol: development and validation of the 4C Mortality Score BMJ 2020; 370 doi: https://doi.org/10.1136/bmj.m3339
